# Role of human organic cation transporter-1 (OCT-1/SLC22A1) in modulating the response to metformin in patients with type 2 diabetes

**DOI:** 10.1186/s12902-022-01033-3

**Published:** 2022-05-26

**Authors:** Fizalah Kawoosa, Zafar A. Shah, Shariq R. Masoodi, Asif Amin, Roohi Rasool, Khalid M. Fazili, Abid Hamid Dar, Asif Lone, Samir ul Bashir

**Affiliations:** 1 Department of Immunology and Molecular Medicine, Sher-I-Kashmir Institute of Medical Sciences, Srinagar, Jammu and Kashmir 190011 India; 2 Department of Endocrinology, Sher-I-Kashmir Institute of Medical Sciences, Soura, Srinagar, Jammu and Kashmir 190011 India; 3 Department of Biotechnology, University of Kashmir, Srinagar, Jammu and Kashmir 190006 India; 4 Department of Biotechnology, Central University of Kashmir, Ganderbal, Jammu and Kashmir 191201 India; 5 Department of Biochemistry, Deshbandhu College, University of Delhi, Delhi, 110019 India; 6 Department of Chemistry, University of Northern British Columbia, Prince George, Canada

**Keywords:** Organic cation transporter 1(OCT-1/SLC22A1), Metformin, AMPK

## Abstract

**Background:**

Organic cation transporter 1 primarily governs the action of metformin in the liver. There are considerable inter-individual variations in metformin response. In light of this, it is crucial to obtain a greater understanding of the influence of OCT1 expression or polymorphism in the context of variable responses elicited by metformin treatment.

**Results:**

We observed that the variable response to metformin in the responders and non-responders is independent of isoform variation and mRNA expression of OCT-1. We also observed an insignificant difference in the serum metformin levels of the patient groups. Further, molecular docking provided us with an insight into the hotspot regions of OCT-1 for metformin binding. Genotyping of these regions revealed SNPs 156T>C and 1222A>G in both the groups, while as 181C>T and 1201G>A were found only in non-responders. The 181T>C and 1222A>G changes were further found to alter OCT-1 structure in silico and affect metformin transport in vitro which was illustrated by their effect on the activation of AMPK, the marker for metformin activity.

**Conclusion:**

Taken together, our results corroborate the role of OCT-1 in the transport of metformin and also point at OCT1 genetic variations possibly affecting the transport of metformin into the cells and hence its subsequent action in responders and non-responders.

**Supplementary Information:**

The online version contains supplementary material available at 10.1186/s12902-022-01033-3.

## Introduction

SLC22A1/OCT-1 is one of the main hepatic-uptake transporters positioned on the sinusoidal membrane of hepatocytes. The human OCT-1 gene has 11 exons and 10 introns. OCT-1 has four isoforms, a long-form that has been reported to be functionally active and three shorter forms that are splice variants of the gene [1]. OCT-1 mediates the uptake, distribution, and efflux of cationic metabolites and drugs in the liver. A broad variety of drugs used in medicine are organic cations, thus the existence of genetic variants in OCT-1 has significant clinical implications in human pharmacology. As an uptake transporter, OCT-1 plays an important role in the efficacy of metformin [2]. Metformin is an insulin-sensitizing agent with effective antihyperglycemic properties. However, there are reports of approximately 35–40% of patients receiving the drug being incapable of achieving controlled levels of fasting blood glucose and hence pointing out that the glycemic response to metformin is variable [3–5]. Polymorphisms in the OCT-1 gene could affect metformin activity by influencing its liver uptake, which in turn could influence the efficacy of the drug [6, 7]. However, there are ambiguous reports on the association between variants of OCT-1 and antihyperglycemic response to metformin [8]. The importance and role of the OCT's/SLCs and their genetic variations in metformin metabolism suggest that additional studies are required to scrutinize the genetic variations and their relationship with metformin response in different ethnic populations.

In general, the hypothesis laid down for this study is based on the consequential variability of drug response apropos of polymorphism and gene expression/isoform variation in the OCT1 gene. This study was aimed at identifying the variability in the OCT1 gene and the interindividual differences in response to the drug. This study could provide us with a basic picture of the responder, non-responder ratio in our population as no such study has been done in our region to date. Also, this was a pilot study carried out on newly diagnosed type 2 diabetes patients that could be recruited for over 1 year only. It was observed that the response to metformin in these patients was independent of isoform variation and mRNA expression of OCT-1, however, some changes in exon 1 and exon 7 were found to have a profound effect on metformin activity.

## Materials and methods

### Materials

For PCR-based work, materials like PCR buffer and Taq polymerase were purchased from Sigma Aldrich, USA, and dNTPs were from Fermentas, USA. For expression studies, materials like cDNA synthesis kit, SYBR green, and ROX solution were purchased from Fermentas, USA. RNA later was purchased from Sigma Aldrich, USA. For cell culture studies, DMEM, FBS, and metformin were purchased from Sigma Aldrich, USA. LB agar and LB broth were purchased from Himedia; DPnI from Thermo scientific; A769962 from Abcam; Q5 polymerase from New England Biolabs and Plasmid extraction kit from Qiagen. For western blot analysis, phospho-AMPK rabbit monoclonal antibody, total AMPK rabbit monoclonal antibody, and beta-actin rabbit monoclonal antibody were purchased from CST, USA. The anti-rabbit IR DYE 800 antibody was purchased from Li-COR Biosciences, USA.

### Study subjects and sample storage

A total of 41 patients clinically diagnosed with type 2 diabetes mellitus were recruited for this study. They were treated with metformin (500-1000 mg/day) for 3 months in the Department of Endocrinology, Sher-I-Kashmir Institute of Medical Sciences, Soura, Srinagar, Kashmir. Based on the response to metformin, patients were classified into two groups: responder group (decrease in HbA1c levels by more than 1% from the baseline i.e. 5.7%) and non-responder group (decrease in HbA1c levels less than 1% from the baseline). Ten liver tissues and their respective blood samples were obtained from the Department of Surgical Gastroenterology, Sher-I-Kashmir Institute of Medical Sciences, Soura, Srinagar, Kashmir.

5 ml peripheral blood samples were collected from responders and non-responders. Out of this quantity, 3 ml was transferred to EDTA vials (200 μl of 0.5 M, pH = 8.0) for RNA and DNA extraction. The rest of the 2 ml blood was used for serum separation and the serum was stored at -80 °C for further analysis. Liver tissues were stored in RNA later at 4 °C overnight and then transferred to -80 °C till further use.

### Isoform and expression analysis of OCT-1 mRNA

Isoform analysis of OCT-1 was carried out using reverse transcription PCR. 1 µg of RNA (extracted by TRIzol method) was reverse transcribed to cDNA using a first-strand cDNA synthesis kit (Thermo scientific). The isoform analysis for the liver tissue and their corresponding blood samples as well as across the patient groups was carried out by setting a PCR reaction using 10X PCR buffer, 10 mM dNTPs, 5U/µl of Taq polymerase, and 10 µM primers for wild OCT-1, isoform-1, isoform-2 and isoform-3 respectively (Table 1S). The PCR products were resolved by 2% agarose gel electrophoresis, ethidium bromide-stained specific bands were visualized under UV light and photographed. They were further sent for sequencing to Macrogen Inc. Korea.

**Table 1:** Genotype distribution and allele frequencies of OCT-1 polymorphisms in responders and non-responders

Genotype	Responder (*n* = 25)	Non-responder (*n* = 16)	*P*-value
**OCT-1 (156 T > C)**			
TT	15 (60.0%)	9 (56.0%)	> 0.05
TC	10 (40.0%)	7 (44.0%)	
**Alleles**			
T	40 (80%)	25 (78.125%)	> 0.05
C	10 (20%)	7 (21.875%)	
**OCT-1 (181 C > T)**			
CC	25 (100%)	6 (37.5%)	** < 0.001**
CT	0	10 (62.5%)	
**Alleles**			
C	50 (100%)	22 (68.75%)	** < 0.001**
T	0 (0%)	10 (31.25%)	
**OCT-1 (1201 G > A)**			
GG	25 (100%)	12(75.0%)	** < 0.05**
GA	0	4 (25%)	
**Alleles**			
G	50 (100%)	28 (87.5%)	** < 0.05**
A	0 (0%)	4 (12.5%)	
**OCT-1 (1222A > G)**			
AA	0	2 (12.5%)	** < 0.05**
AG	05 (20.0%)	9 (56.25%)	
GG	20 (80.0%)	5 (31.25%)	
**Alleles**			
A	5 (10%)	13 (40.625%)	** < 0.05**
G	45 (90%)	19 (59.375%)	

For the expression analysis of OCT-1 mRNA between patient groups (responders and non-responders), real-time quantitative PCR was utilized. The real-time PCR was performed with a light cycler PCR instrument (Applied Biosystems) in a 96 well plate in triplicates using SYBR Green master mix (Fermentas). The ct values of each patient were normalized to GAPDH using the SYBR Green-based comparative CT method (2^−∆∆CT^). For comparison, OCT-1 expression in blood was calculated using responder sample expression as a reference control. Primers used for qRT-PCR are listed in Table 2S.

### Molecular docking

Molecular docking was carried out to analyze the interaction of metformin with OCT-1. OCT-1 protein structure was not available and therefore was generated by template modeling using Swiss Model online software and by generating the best fit structure using iTasser. The structure of metformin was acquired from PubChem. SwissDock and Autodock4 were employed for docking metformin on the OCT1 structure. Lamarckian genetic algorithm with local search (GALS) was used as a search engine, with a total of 100 runs. The dock run was programmed to score for blind docking with a minimum forced planner. Cluster analysis was performed on the docked results using an RMS tolerance of 2.0 Å. Finally, the more energetically favorable cluster poses were evaluated by using USCF Chimera software.

### Polymorphic/Mutational analysis

The exons 1 and 7 of the OCT-1 gene were selected for polymorphic/mutational analysis after carrying out docking analysis. Primers were designed against these exons (Table 3S) and a PCR reaction was set up using 10 µM of these primers, DNA (250 µg/µl) from both patient groups, 10X PCR buffer, 10 mM dNTP, and 5U/µl of Taq polymerase for amplification of these exons. The amplified samples (25 µl each) were sent for whole exon sequencing which was done using an ABI prism at Macrogen Inc.Korea. Sequence scanner software (Bioedit, USA) was used for comparing sequences between the original and the chromatogram sequences.

### Molecular dynamics simulations

Molecular Dynamics simulations (MDS) were performed on the wild-type protein and mutants using the GROMACS 4.6 platform and the GROMOS CHARMM force field [9]. Before MD simulations, all three complexes were treated to an energy minimization approach, and their positions were restricted in NVT and NPT ensembles at 300 k for 100 ps. Both NVT and NPT ensembles employed Berendsen's coupling approach. The SHAKE algorithm, as well as the Particle mesh Ewald approach for long-range electrostatics, a 14 cut-off for van der Waals interactions, and a 12 cut-off for Coulomb interactions with updates every two steps, were used to confine bond lengths. After MD simulations, the potential of each generated trajectory was thoroughly examined. The root-mean-square deviation (RMSD), root-mean-square fluctuation (RMSF), the radius of gyration (Rg), and the number of H bonds formed between the ligand and proteins were calculated using the GROMACS tools g rms, g rmsf, g hbond, and g gyrate. To see whether the systems obey NVT or NPT ensemble throughout the simulation, the differences in kinetic, potential, and total energy, pressure, and temperature were estimated as a function of simulation time. To understand the difference in ligand–protein stability, the total number of hydrogen bonds was computed. To determine the solvent-accessible surface area, SASA was used. The trajectories were evaluated with the GROMACS distribution's tools. All of the graphs were created with XMgrace.

### In-vitro functional/cellular assays

The sequence analysis of the clinical samples was followed by PCR-based mutagenesis in cell lines. This was followed by the transfection of mutant constructs into a suitable model cell line (HepG2). Functional assays were carried out on transfected cells that include the downstream effector signal molecule analysis viz quantification of activated AMPK by western blot.

### Site-directed mutagenesis (SDM) and transformation of DH5α cells with OCT-1 variants

We received the OCT-1 wild plasmid as a kind gift from Kathleen M. Giacomini, Department of Biopharmaceutical Sciences, University of California, San Francisco. The concentration of the plasmid was 140.1 ng/ul and the reference vector/backbone was pcDNA5. The variants R61C (181C > T), G401S (1201G > A), and M408V (1222A > G) were constructed by site-directed mutagenesis using the wild plasmid as a template and setting up PCR reactions using primers listed in Table 4S. High fidelity Q _5_ polymerase was used to minimize the incorporation of wrong bases. The PCR product was run on 1% agarose gel to confirm the successful amplification. The mutant constructs of OCT-1 were transformed into DH5α cells separately. The overnight cultured broth was subjected to plasmid extraction using the QIA Prep^R^ Spin miniprep kit from QIAGEN.

### Transfection and drug treatment

HepG2 liver carcinoma cells were cultured in Dulbecco’s modified Eagles medium (DMEM) supplemented with 10% (v/v) fetal bovine serum, 50 μg /ml of penicillin, and 0.1 mg/ml streptomycin. The cells were kept in a humidified atmosphere of 5% CO2/95% air at 37 °C. Further, the HEPG2 cells were transiently transfected with the vector, wild, and mutant OCT-1 constructs using polyethyleneimine (PEI). They were then treated with 20 µM of metformin and 100 µM A769962. Finally, the cells were processed for RNA extraction, and protein extraction, while the expression studies were monitored by western blotting.

### Western blotting

Western blotting was used for analyzing pAMPK expression in metformin-treated and untreated HEPG2 cells expressing vehicle control, reference OCT-1 and OCT-1 mutant constructs. Briefly, cells were harvested, and lysed in RIPA buffer for protein extraction. The protein concentrations were determined by the Bradford method. Equal amounts of protein from whole-cell lysates were separated on SDS–polyacrylamide gel (10–12%) and then electrophoretically transferred onto the PVDF membrane. Blots were incubated with primary antibodies against phospho AMPK/pAMPK (CST), total AMPK/tAMPK (CST), and β-actin (CST) at 4 °C overnight The dilutions used were 1:1000 for pAMPK, 1:1000 for tAMPK and 1:5000 for β-actin respectively. After washing, the blots were incubated with appropriate IR-tagged secondary antibodies (Licor Biosciences) used at a dilution of 1:10,000 for 1 h at room temperature. The blots were then scanned in an infrared image scanner (Licor Biosciences). (The images of full-length blots are included in Supplementary file S2. However, we could not retrieve a full-length picture of a blot comprising the middle panel of Fig. 5 as the image was saved as such in the Licor imager).

### Estimation of serum metformin levels

The samples used for the analysis were the serum samples obtained from patients (10 responders and 10 non-responders) who were on 1000 mg of metformin. The analysis was performed on a Shimadzu UFLC chromatographic system equipped with an LC-20AD solvent delivery pump, PDA SPD M-20A detector, DGU-20A degasser, SL-20AHT autosampler, and CTO-10AS column oven. A Merck high resolution chromolith RP-18e column (100 mm × 4.6 mm, 5um particles; temperature set to 25 °C) was used and the mobile phase consisted of 80% acetonitrile: 20% water containing 0.1% formic acid. Analyses were run at a flow rate of 0.5 ml/min. The peak height ratios of six calibration standards (0.02–0.8 µg/µl for responders and 0.01–0.4 µg/µl for non-responders) were used to establish the standard curves and the standard curves were linear (r^2^ ≥ 0.999 for responders and r^2^ ≥ 0.997 for non-responders).

### Statistical analysis

The software package SPSS (Statistical Package for the Social Sciences) was used for statistical analysis. Student-t test, direct gene count method, Chi-square test, Fischer's exact test, and multivariate analysis were used wherever applicable. Differences were considered statistically significant when the '*p*—value' was < 0.05.

## Results

### Clinical characteristics of study subjects

In this study, subjects were divided into two groups: responders (*n* = 25) and non-responders (*n* = 16). The groups did not differ significantly in age (40.04 ± 9.19 in the responder group, 46.81 ± 8.93 in the non-responder group, *p* > 0.05). Of all the participants, 15 were males and 10 were females in responders whereas 8 were male and 8 were female in non-responders. Values of the study parameters based on responders and non-responders are presented in Table 5S and Table 6S. As shown in the tables, the parameters like FBS, PPG, TC, uric acid, creatinine, PPI intake, metformin dosage, and HbA1c levels varied significantly between responders and non-responders. To assess the effects of potential confounding variables like age, PPI intake, hyperuricemia, etc. on metformin response, multivariate analysis was performed (Table 7S). It was observed that the response to metformin is independent of all these factors, further strengthening our hypothesis that the variation in metformin response is primarily due to changes in OCT-1.

### Isoform analysis and mRNA quantification of OCT-1

**Fig. 1:**
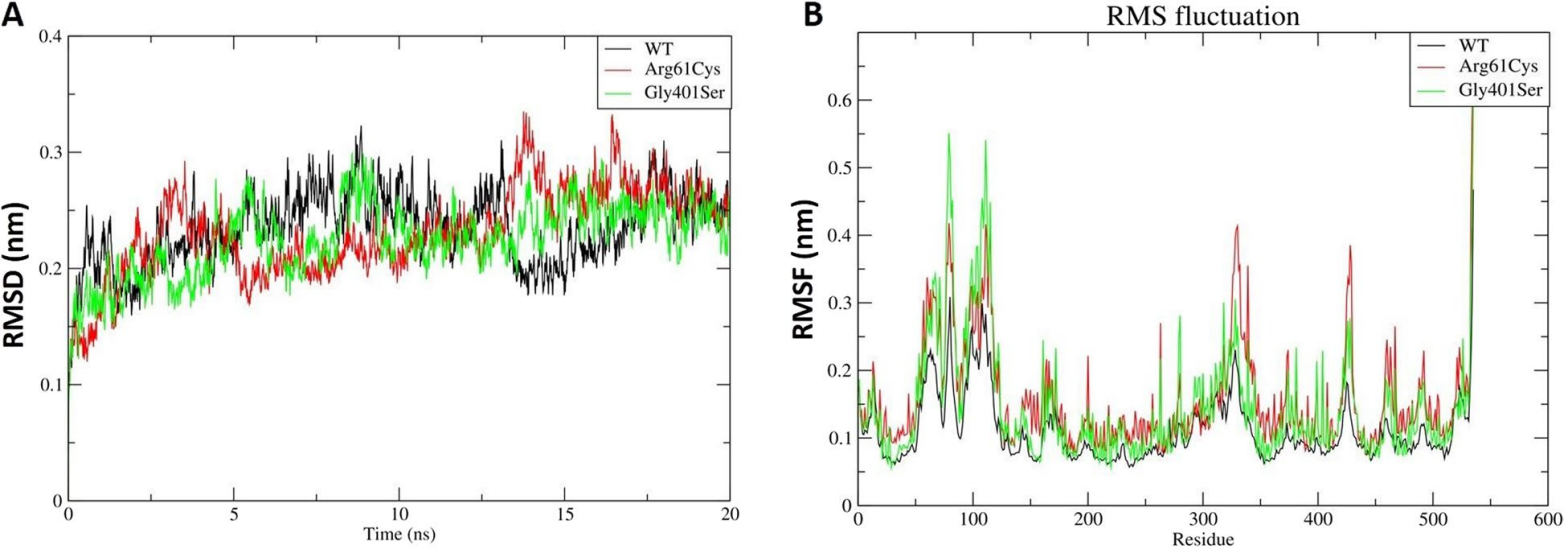
MD simulations of wild-type (black) (p.Arg61Cys) (red) and (p.Gly401Ser) (green) variants structures. **A** Backbone RMSD for wild type and variant structures at 300 k plotted as function of time. **B** Comparative RMSF plot showing fluctuations and wild-type and variant structures along the protein stretch

**Fig. 2:**
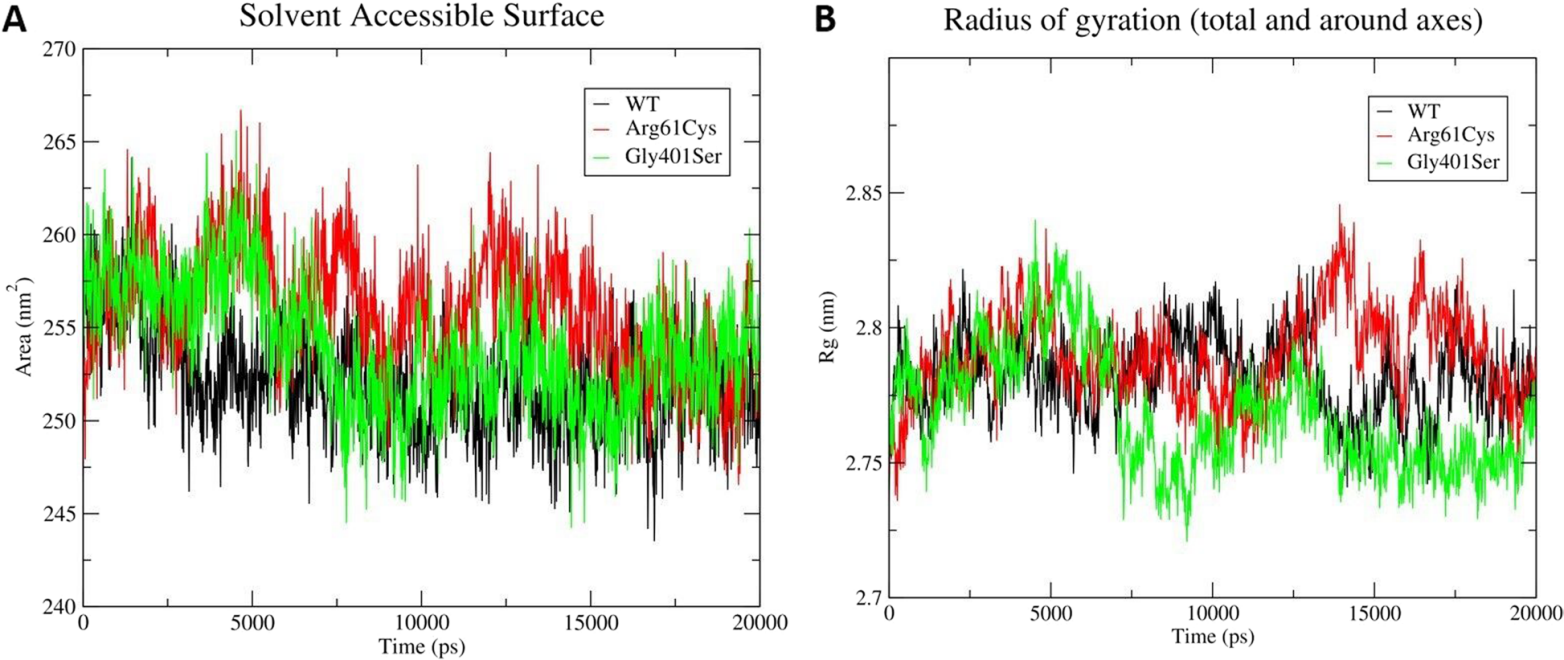
Graph depicting relative trend in (A), surface area and (B), radius of gyration (Rg) of wild-type (black), (p.Arg61Cys) (red) and (p.Gly401Ser) (green) variants structures, during 20 ns MD simulation

**Fig. 3:**
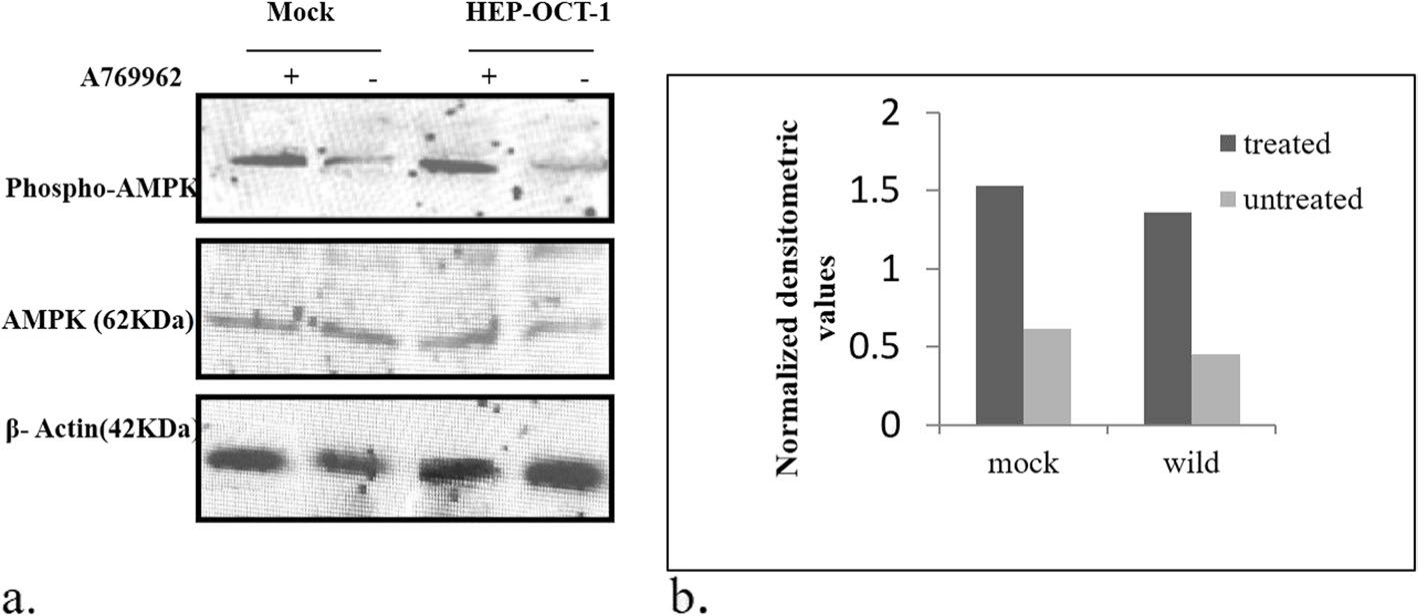
a Effect of A769962 on AMPK phosphorylation in mocks vs wild OCT-1 transfected HepG2 cells: Representative ummunoblots showing the change in pAMPK expression by the presence/absence of A769962 in cells expressing vehicle control and OCT-1. The lower panel represents the effect of the same on the expression of β–actin protein used as a loading control. b Graph showing normalised densitometric values for the A769962 treated and untreated mock and wild type cells

**Fig. 4:**
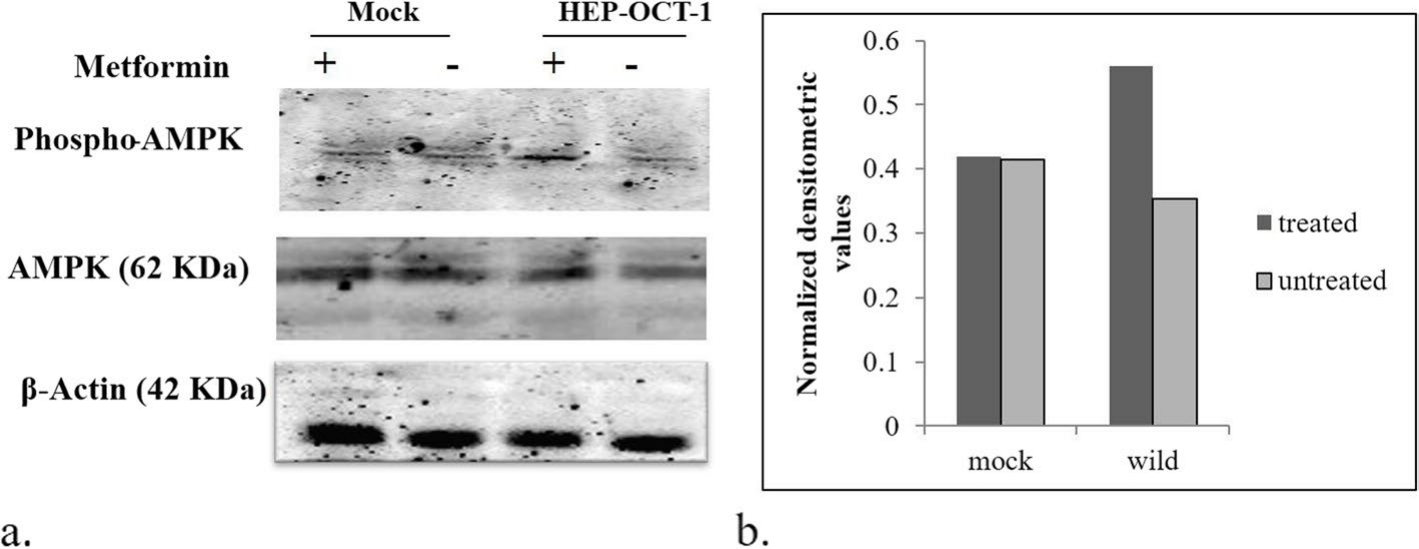
a Effect of metformin on AMPK phosphorylation in mocks vs wild OCT-1 transfected HepG2 cells: Representative ummunoblots showing the change in pAMPK expression by the presence/absence of metformin in cells expressing vehicle control and OCT-1. The lower panel represents the effect of the same on the expression of β–actin protein used as a loading control. b Graph showing normalised densitometric values for the metformin treated and untreated mock and wild type cells

**Fig. 5:**
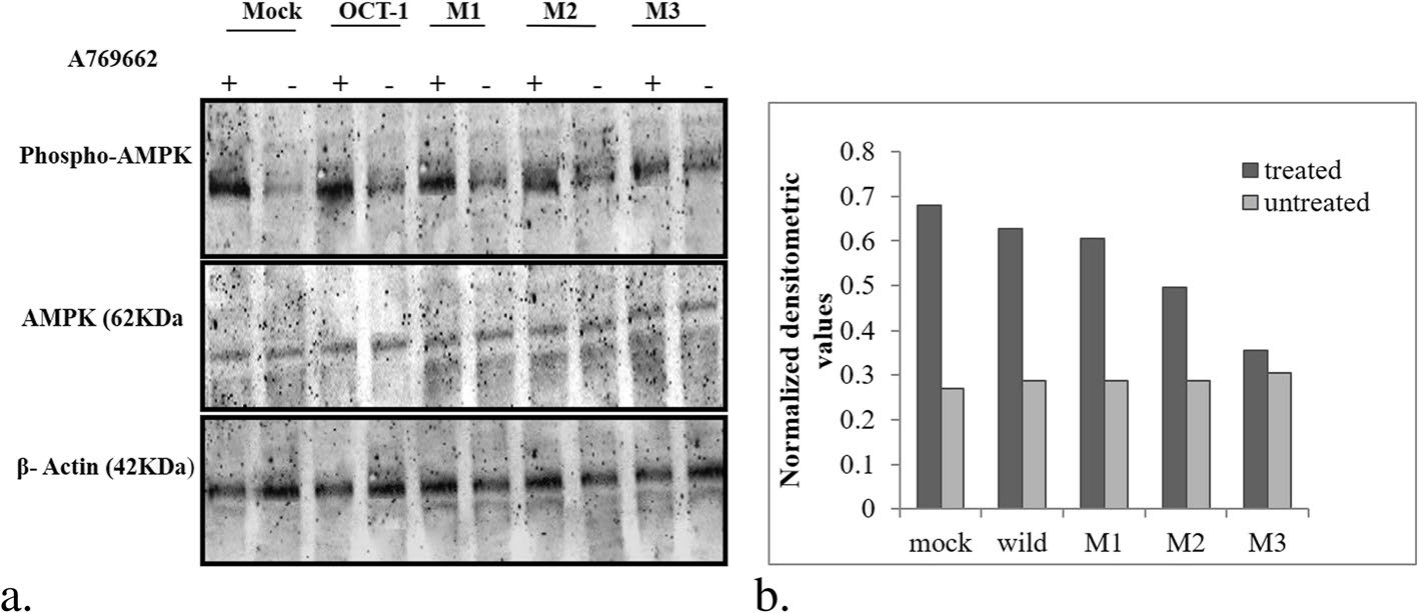
a Effect of A769962 on AMPK phosphorylation in mock, reference OCT-1 and mutant transfects: Representative ummunoblots showing the change in pAMPK expression by the presence/absence of A769962 in HepG2 cells expressing vehicle control, reference OCT-1 and mutant OCT-1 constructs. The lower panel represents the effect of the same on the expression of β–actin protein used as a loading control. M1: 181C > T; M2; 1201G > A; M3: 1222A > G. b Graph showing normalised densitometric values for the A769962 treated and untreated mock, wild type cells and mutant cells

**Fig. 6:**
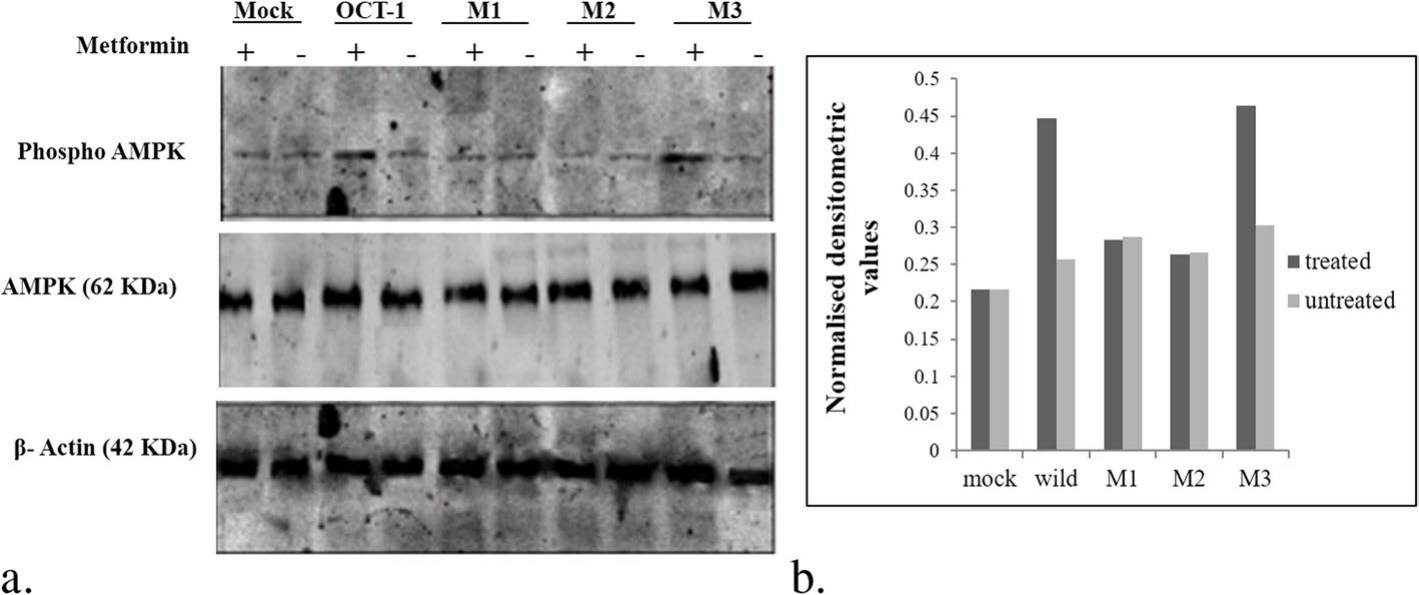
a Effect of metformin on AMPK phosphorylation in mock, reference OCT-1 and mutant transfects: Representative ummunoblots showing the change in pAMPK expression by the presence/absence of metformin in HepG2 cells expressing vehicle control, reference OCT-1 and mutant OCT-1 constructs. The lower panel represents the effect of the same on the expression of β–actin protein used as a loading control. M1: 181C > T; M2; 1201G > A; M3: 1222A > G. b Graph showing normalised densitometric values for the metformin- treated and untreated mock, wild type cells and mutant cells

We analyzed the pattern of OCT-1 isoforms in the liver tissue and corresponding blood sample as well as across the patient groups using specific primers against these isoforms. We observed that the pattern of isoforms was similar in both liver tissues and their respective blood samples as well as across the two patient groups. The full-length OCT-1, isoform-1 and isoform-3 were found in all the samples whereas isoform-2 was not present in any of these (Figs. 1S, 2Sa, and 2Sb). The partial electropherograms of the PCR products are shown in Figs. 3Sa, 3Sb, and 3Sc. To substantiate our results, we aligned the sequences of the PCR products with the full-length OCT-1, isoform-1, isoform-2, and isoform-3 cDNA sequences that were retrieved from the database (GeneCards) (Figs. 4S, 5S, and 6S), thus validating our results further. On expression analysis of OCT-1 between the two patient groups, the fold change for OCT-1 expression was found to be insignificant with a *p*—value being > 0.05 (Fig. 7S).

### Molecular docking of metformin on OCT-1

The structure of OCT-1 protein (Figs. 8SA, and 8SB) and metformin (Fig. 8SC) were docked to form a complex. The docked complex of metformin and OCT-1 protein revealed substantial information on the possible hotspot interactive pockets of OCT-1 protein. Some of the top docked complexes having estimated lower delta-G were distributed in clusters all along the OCT-1 structure (Fig. 8SD-E). Out of four, three clusters (Fig. 8SF) showed adequate hydrogen bonding along with a favorable energy score. The total energy, delta-G, and H-bond number are listed for only those complexes having two hydrogen bonds shared between OCT-1 and metformin (Table 8S). Cluster 1 (Fig. 8SG-H) and Cluster 2 (Fig. 8SI-J) represent the metformin binding to amino acid stretches located in the extracellular regions of OCT-1 protein (ARG61, GLN60, TYR256 ALA257, and PRO259) with varying orientation. Cluster 3(8SK-L) reveals metformin binding in the inner pocket of OCT-1 channel protein (GLY477 and inner pore helices of exon-1 and 7). All those interactions featuring on the outer face of OCT-1 which in the physiological state would be in close contact with the lipid bilayer, were not considered as best fit irrespective of their stable interaction.

### Genotyping of exon-1 and exon-7

Molecular docking revealed the hotspot regions for metformin binding on OCT-1 protein and these regions majorly spanned exon 1 and exon 7. Sequencing of the exon-1 and exon-7 PCR products (Figs. 9S and 10S) revealed the presence of 156 T > C (rs1867351) and 181C > T (rs12208357) change in exon-1 and 1201G > A (rs34130495) and 1222A > G (rs628031) change in exon-7. The partial electropherograms are shown in Figs. 11SA-D. The distribution of various genotypes of OCT-1 in study groups and the association of genotypes and the alleles with them are shown in Table 1. On analyzing the data statistically, it was observed that the 181 CT, and 1201 GA genotypes were significantly associated with non-responders (*p* < 0.001, < 0.05 respectively) whereas the genotype 1222 GG was found to be significantly associated with the responders (*p* < 0.05). As far as allele frequencies are concerned, 181 T allele and 1201A were found to be significantly associated with the non-responders (*p* < 0.001 and *p* < 0.05 respectively). However, the 1222 G allele was found to be significantly associated with responders (*p* < 0.05).

### Structural and conformational dynamics of OCT-1 (p.Arg61Cys) and (p.Gly401Ser) variants

To assess the effect of the reported SNPs on the OCT-1 protein structure and conformation, molecular dynamic simulation of 181C > T (p.Arg61Cys) and 1201G > A (p.Gly401Ser) variants identified in non-responders leading to Arginine-Cystine and Glycine-Serine substitution was carried out. The energy-minimized structures were subjected to two independent 20 ns runs under the GROMOS CHARMM force field (Supplementary Video S1: Animation). The root mean square deviation (RMSD) of the three structures was calculated over time using g rms tool. All the three runs were stable over the period of 20 ns with maximum fluctuations observed at ~ 14 and ~ 17 ns in (p.Arg61Cys) variant (red) and ~ 14 ns in (p.Gly401Ser) variant (green) structures respectively (Fig. 1). RMSD trajectory value in the wild-type protein structure ranged from 0.08 to 0.32 nm while that of the (p.Arg61Cys) variant structure fell between 0.08 and 0.34 nm. Up to 5000 ps, this mutant protein structure showed an increase in RMSD reaching 0.29 nm followed by a decrease in RMSD till 13,000 ps. After 13,000 ps, an increase in RMSD to 0.34 nm was again observed. The average RMSD trajectory value in the (p.Gly401Ser) variant spans from 0.08 to 0.30 nm. The RMSD of this variant remains nearly consistent. At 3000 ps and 6600 ps, minor differences were observed in the structure of this protein. At 8500 ps and 14,000 ps, an increase in the RMSD value of this structure was observed, following which the RMSD trajectory remains constant until the simulation time period ends.

To establish whether mutations impact the dynamic behavior of amino acid residues, RMSF values of wild-type and mutant structures were computed using the g rmsf tool. The RMSF is used to describe flexibility differences among residues in relation to the average MD simulation conformation. The wild-type protein structure was found to exhibit an RMSF range of 0.05 to 0.30 nm with residues between 50 and 125 showing significant variability (Fig. 1). With a fluctuation of 0.30 nm, the largest RMSF fluctuation was observed at residue number 79. In contrast to wild-type protein, (p.Arg61Cys) variant structure depicted an RMSF range of 0.05 to 0.42 nm. When comparing this variant’s protein residues to the wild-type structure, the results depict an increase in overall fluctuation. All residues showed high RMSF when compared to wild-type RMSF. (p.Gly401Ser) variant exhibited the highest RMSD fluctuations during the simulation between 50 and 125 residues when compared to the other two structures with RMSF values ranging from 0.07 to 0.55 nm.

Next solvent-accessibility of the wild-type and variant structures was computed using the SASA tool. Among the three structures studied, the (p.Arg61Cys) variant showed the highest SASA followed by the (p.Gly401Ser) variant (Fig. 2). The wild-type structure exhibited the least SASA during the simulation. The findings point at an overall expansion in the protein due to the 181C > T (p.Arg61Cys) and 1201G > A (p.Gly401Ser) variation. Finally, the Radius of Gyration (Rg) was calculated to determine the amounts of compaction in the original and variant structures. It was observed that there is a noticeable increase in the Rg value of both the (p.Arg61Cys) and (p.Gly401Ser) variants in comparison to the wild-type structure (Fig. 2), thereby suggesting that both variations lead to the expanded protein structure.

### In-vitro functional assays

The wild-type OCT-1 gene was subjected to site-directed mutagenesis to create the mutant constructs having changes that we obtained after genotyping of patient samples. These mutant constructs generated were confirmed by sequencing of the PCR products (Fig. 12S, 13S).

### Expression analysis

AMPK functions as a ser/thr kinase whose activity is known to be altered by metformin. Owing to this fact, we choose to study the transport of metformin via OCT-1 into the cells and considered activation of AMPK as an indicator of metformin activity. Therefore, protein expression of phospho-AMPK and total AMPK was checked to analyze the effect of the mutations in OCT-1 on the transport of metformin. As metformin is known to activate AMPK, therefore, we carried out proteomic studies for the same. We observed that mutant 181C > T and mutant 1201G > A had a profound effect on AMPK activation/ phosphorylation. The activation of AMPK was indicated by increased expression of phospho-AMPK and vice versa. We found that variants 181 C > T and 1201 G > A show decreased expression of phospho-AMPK when compared to the wild OCT-1 counterpart. However, the expression of total AMPK was not affected by any of these changes in OCT-1. Before the metformin treatment and subsequent experiments, the effect of the AMPK activator (A769962) was also observed in these cells, to confirm that the effects observed for metformin-treated cells were exclusively due to metformin only (Fig. 3a, 3b; Fig. 4a, 4b; Fig. 5a, 5b; and Fig. 6a, 6b).

### Estimation of serum metformin levels in the study subjects

We performed HPLC analysis to evaluate the levels of metformin in the serum of patients from both groups. The mean ± SD values for responders and non-responders were 0.11 ± 0.02 and 0.14 ± 0.06 respectively (Table 9S). Although there was a slight increase in the levels of metformin in the serum of non-responders as compared to the responders, it was found to be non-significant (*p* > 0.05).

## Discussion

Our study was a pilot study, that focussed on patients suffering from diabetes to find the underlying difference between the cohort of patients that were put on metformin medication but were unable to respond to the treatment viz a viz patients who responded to metformin therapy. These patients were divided into responders and non-responders based on their response to metformin which was assessed by the decrease in HbA1c levels in them. HbA1c level has been assessed as a marker of treatment response in patients with diabetes in many studies [10–12]. In the present study, a reduction of 1% in HbA1c has been deemed a response to metformin therapy. Previous studies have shown that oral antidiabetic drugs effectively decrease HbA1c levels by 0.5–1.5% [13]. Our study indicated that the mean change (post vs pre-treatment) in HbA1c was -1.94 ± 1 among responders, whereas the value was 1.23 ± 1.33 in non-responders (Table 6S). This result demonstrates that the greater proportion of the decrease in HbA1c levels in all patients after metformin therapy was associated with responders, suggesting that metformin response could be important in evaluating HbA1c as a key indicator in monitoring long-term glycemic control. In addition to HbA1c levels, various other clinical parameters considered for our study included weight, age, BMI, fasting glucose levels, HDL, LDL levels, creatinine, uric acid levels, PPI intake, statin intake, etc. The comparison of the clinical parameters between the two groups was carried out to further understand the differences that might play important roles in differentiating between these two groups of patients (Table 5S). We found a significant difference in fasting blood glucose and postprandial glucose levels between the two groups. This further strengthened our basis of differentiation between the two sets of patients. The difference in the FBS and PPG levels between responders and non-responders indicated that metformin is not quite effective in lowering blood sugar levels in non-responders. Similar differences in blood sugar levels between the two groups were also reported in some other studies [14–16]**.** Further, we also found a higher number of people taking PPI in the non-responder group (*p* < 0.05). Our observation is further supported by the study that identified PPIs as an important drug class inhibiting OCT-mediated metformin transport [17]. In our study, when we compared the metformin dosage between the two groups, we found that the average metformin dosage was higher for non-responders than responders (*p* = 0.001). This further clarifies that non-responders do not sufficiently respond to metformin even at higher doses of metformin. There is an increase in HbA1c levels of non-responders as compared to responders, which also comes as a part of studies elsewhere wherein it has been reported that higher doses of metformin lead to better glycemic control in patients with diabetes [18, 19]. To substantiate that none of the clinical factors were affecting the response to metformin, we performed a multivariate analysis. The results of the analysis showed that metformin response was independent of all the potential confounding factors further implicating the role of OCT-1 in metformin response variability (Table 7S).

Further, we studied the isoform pattern between responders and non-responders. We hypothesized that the difference in the response between responders and non-responders might be due to the difference in the type of isoforms between the two groups that could have led to variation in the response due to differential uptake of metformin. Since the four isoforms of OCT-1 were reported from liver tissue [1], we initially looked at the expression of OCT-1 isoforms in the liver and blood samples of the same patient. Convincingly, we observed identical isoforms present in the liver and blood coming from the same patient. The identity of isoforms was confirmed by sequencing. However, our results differed from that of Hayer et al. as we found three isoforms (full-length OCT-1, isoform 1, and isoform 3) in liver samples as opposed to two (full-length OCT-1 and isoform 1) found in their study [1]. The alignment of the sequences (obtained after sequencing the amplification products of full-length OCT-1, isoform-1, and isoform-3) with the full length and isoform sequences of OCT-1 present in the database (retrieved from Gene Cards) further validated our results (Figs. 4S, 5S and 6S). Hence, we inferred that the isoform variation might not be the cause of the disparity in metformin response. Next, we moved on to expression analysis of wild OCT-1 in both patient sets. We found that there was no significant difference in the expression of OCT-1 between responders and non-responders (Fig. 7S).

To get a clearer picture of varying metformin responses between the two groups of patients we further moved on to analyze the hotspot domains of OCT-1 protein, likely responsible for metformin binding and transport across the cell. We found that among the other metformin binding regions predicted by docking it on OCT-1 protein model, three regions showed a high docking score (Table 5S). Out of these three, two were located in the extracellular domain and one inside the pocket of OCT-1 (Fig. 8SF). The third one i.e. the cluster located in the pocket, besides other interactions, also interacted with the inner pore helix of exon-1 and exon-7. Taking a lead from these observations, we set out to analyze the mutational basis of the extracellular domains of OCT-1 protein majorly spanning through exon-1 and 7. Concomitantly, these regions of the OCT-1 gene have most of the reported SNPs/Mutations affecting either the expression of OCT-1 or its transportability. These exons were amplified and then PCR products were sequenced. We reported SNPs 156 T > C and 181C > T in exon-1; SNPs 1201G > A, 1222A > G in exon-7. The SNPs 156 T > C and 1222A > G were found in both groups of patients although with varying frequencies. However, SNPs 181C > T and 1201G > A were found in non-responders only. Several studies have reported the presence of these and other SNPs in OCT-1. Shu et al. have indicted the role of 1201G > A in reduced transport of metformin *invitro* [2]. A study has reported a reduction of 87% to 98% of transport of various classical but structurally diverse OCT substrates due to this variant [20]. The variant 1222A > G has been shown to affect liver uptake of metformin, and consequently influence the efficacy of metformin [21, 22]. By contrast, an investigation of Iranian [23], Indian [24], Caucasian [25] and Japanese [21, 26] populations showed that there was no association between this polymorphism and metformin responses. A study by Kerb et al. has previously described the functional consequences of 181 C > T variant in vitro using the oocyte expression system and detected a 70% reduction of MPP transport activity compared with the OCT-1 reference sequence [20]. When recombinantly expressed in mammalian human embryonic kidney cells and using metformin as the substrate, this variant showed only 5% transport activity compared with the reference sequence [2]. The effect of 156 T > C on the glycemic response to metformin has been investigated in Han Chinese and Indian populations. No significant effect of the minor C allele has been shown while the T/T genotype exhibited a greater reduction in PPG and HbA1c levels (*P* = 0.020) in the Han Chinese population [27]. We further tried to examine the functional implications of 181C > T and 1201G > A variations on the OCT-1 protein structure and architecture. These variants essentially lead to missense variations in the predictive protein harboring (p.Arg61Cys) and (p.Gly401Ser) variations respectively. When MD trajectories of wild type and both the variants were analyzed for 20 ns, discernable changes in RMSF, SASA and Rg were observed which pointed to overall expansion in the structure of both the variant proteins. Overall, the findings reflect that both the variations do not favor the structural compactness of the protein, which in turn leads to its compromised and aberrated conformation that may impede the OCT-1 function and hence metformin uptake.

We also carried out an estimation of metformin levels in serum by HPLC to find the difference in metformin levels between responders and non-responders. The serum metformin levels were slightly higher in non-responders than responders although the association was statistically insignificant (*p* > 0.05). We next moved on to performing the *invitro* functional assays to examine the effects of the variations reported in our study, on metformin transport. We generated 181 C > T, 1201 G > A, and 1222 A > G mutants using site-directed mutagenesis. These were transfected into HepG2 cells and experiments were set up using metformin and A769962 (AMPK activator). We assessed the effect of metformin in these mutants by looking at the phosphorylation status of pAMPK, which is known to be a functional reporter or marker for metformin activity. While mutant 1222 A > G did not affect the pAMPK levels, 181 C > T and 1201 G > A led to a decrease in pAMPK expression as compared to reference wild-type OCT-1. Concomitant to our results, Shu et al. also reported seven SNPs in OCT-1 including 181 C > T and 1201 G > A variants that exhibit reduced transport of metformin [2]. They concluded that OCT-1 mediates the first step in the response pathway of metformin and the genetic variation in OCT-1 may modulate response to metformin in humans.

## Conclusion

Our study points out the importance of OCT-1 and its variants in the transport of metformin into the cells and hence its subsequent action. Molecular docking analysis revealed the role played by exon-1 and exon-7 in the binding of metformin to OCT-1 The SNPs associated with these hot spot binding regions of OCT-1 were found to affect the transport of metformin in vitro. Our study also concluded that the variation in the metformin response between the two groups of patients i.e. responders and non-responders is independent of isoform variation and mRNA expression of OCT-1 further hinting that the changes in the OCT-1 gene lead to this varied response to metformin in our patient groups. Furthermore, variations in non-responders were observed to oppose the structural compactness of the protein, thus leading to its compromised and aberrated conformation that may impede the OCT-1 function and hence metformin uptake. However, this work was a pilot study and therefore further studies are needed to be carried out on a larger cohort of patients to get a clearer picture of the OCT-1 and metformin relation. Also, further studies are warranted to examine the effects of the reported SNPs on the protein folding, substrate binding, and substrate selectivity of the OCT-1 transporter.

## Supplementary Information

**Additional file 1.**

**Additional file 2.**

**Additional file 3.**

## Data Availability

All data generated or analyzed during this study are included in this published article [and its supplementary information files].

## Abbreviations

AMPK: 5ʹ AMP-activated protein kinase
dNTP: Deoxyribonucleotide triphosphate
EDTA: Ethylene diamine tetra acetic acid
FBS: Fasting blood sugar
GAPDH: Glyceraldehye 3-phosphate dehydrogenase
HPLC: High performance liquid chromatography
HDL: High density lipoprotein
kDa: Kilodalton
LDL: Low density lipoprotein
LC-MS: Liquid chromatography-mass spectrometry
OCT: Organic cation transporter
pAMPK: Phosphorylated AMPK
PPI: Proton pump inhibitor
PPG: Post prandial glucose
PAGE: Polyacrylamide gel electrophoresis
PEI: Polyethylenimine
RIPA: Radioimmunoprecipitation assay buffer
SLC: Solute carrier
SDS: Sodium dodecyl sulphate
SNP: Single nucleotide polymorphism
SDM: Site directed mutagenesis
tAMPK: Total AMPK
