# Supplementary material for: Role of human organic cation transporter-1 (OCT-1/SLC22A1) in modulating the response to metformin in patients with type 2 diabetes
Source: BMC Endocr Disord. 2022 May 26;22:140. doi: 10.1186/s12902-022-01033-3 (PMC9137212; doi:10.1186/s12902-022-01033-3)
Supplement: Supplementary file 1 — Additional file 1. [file 12902_2022_1033_MOESM1_ESM.ppt]

## Slide 1
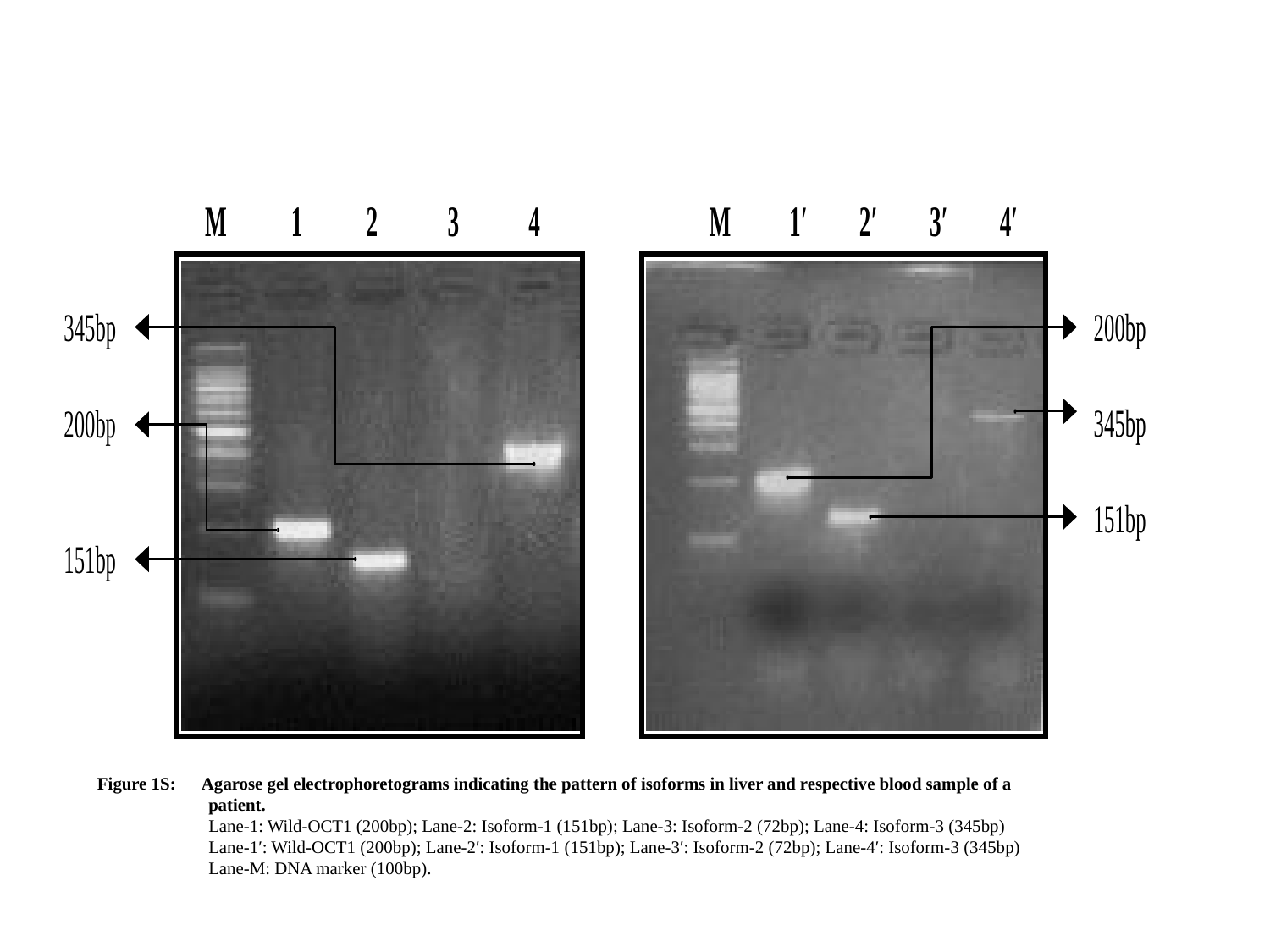

Figure 1S: Agarose gel electrophoretograms indicating the pattern of isoforms in liver and respective blood sample of a
 patient.
 Lane-1: Wild-OCT1 (200bp); Lane-2: Isoform-1 (151bp); Lane-3: Isoform-2 (72bp); Lane-4: Isoform-3 (345bp)
 Lane-1ʹ: Wild-OCT1 (200bp); Lane-2ʹ: Isoform-1 (151bp); Lane-3ʹ: Isoform-2 (72bp); Lane-4ʹ: Isoform-3 (345bp)
 Lane-M: DNA marker (100bp).

## Slide 2
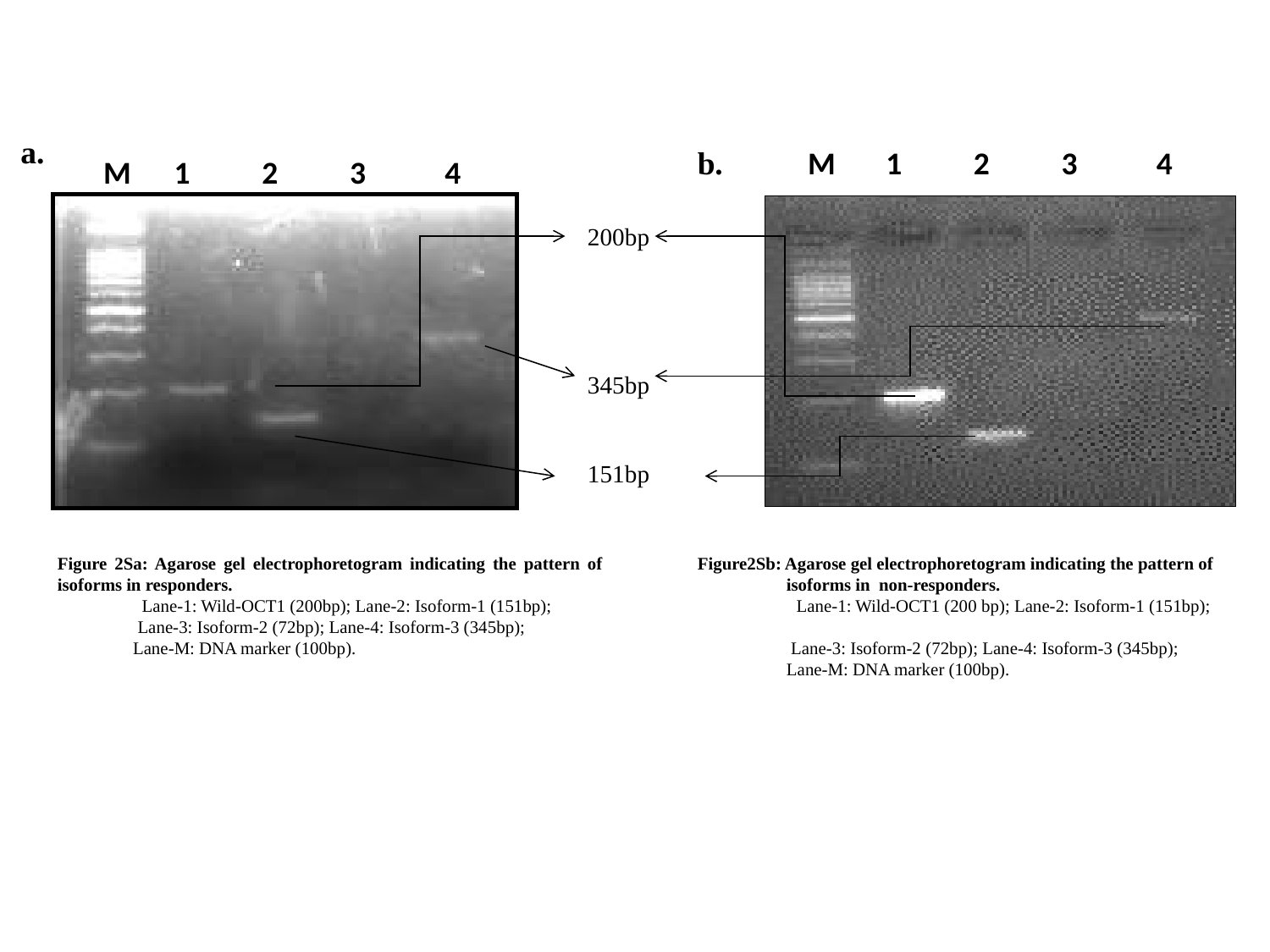

a.
b.
 M 1 2 3 4
 M 1 2 3 4
200bp
345bp
151bp
Figure 2Sa: Agarose gel electrophoretogram indicating the pattern of isoforms in responders.
 Lane-1: Wild-OCT1 (200bp); Lane-2: Isoform-1 (151bp);
 Lane-3: Isoform-2 (72bp); Lane-4: Isoform-3 (345bp);
 Lane-M: DNA marker (100bp).
Figure2Sb: Agarose gel electrophoretogram indicating the pattern of
 isoforms in non-responders.
 Lane-1: Wild-OCT1 (200 bp); Lane-2: Isoform-1 (151bp);
 Lane-3: Isoform-2 (72bp); Lane-4: Isoform-3 (345bp);
 Lane-M: DNA marker (100bp).

## Slide 3
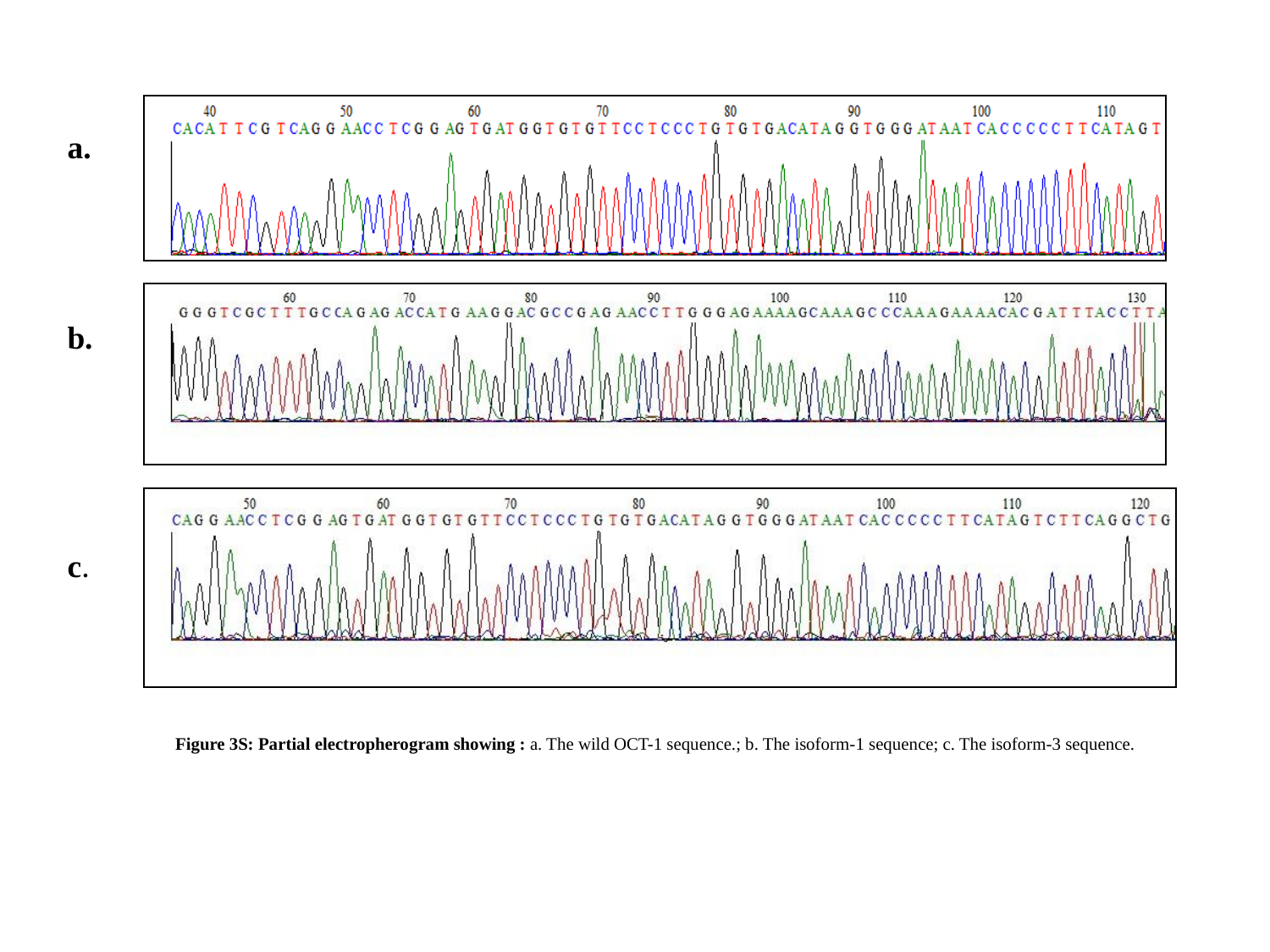

a.
b.
c.
Figure 3S: Partial electropherogram showing : a. The wild OCT-1 sequence.; b. The isoform-1 sequence; c. The isoform-3 sequence.

## Slide 4
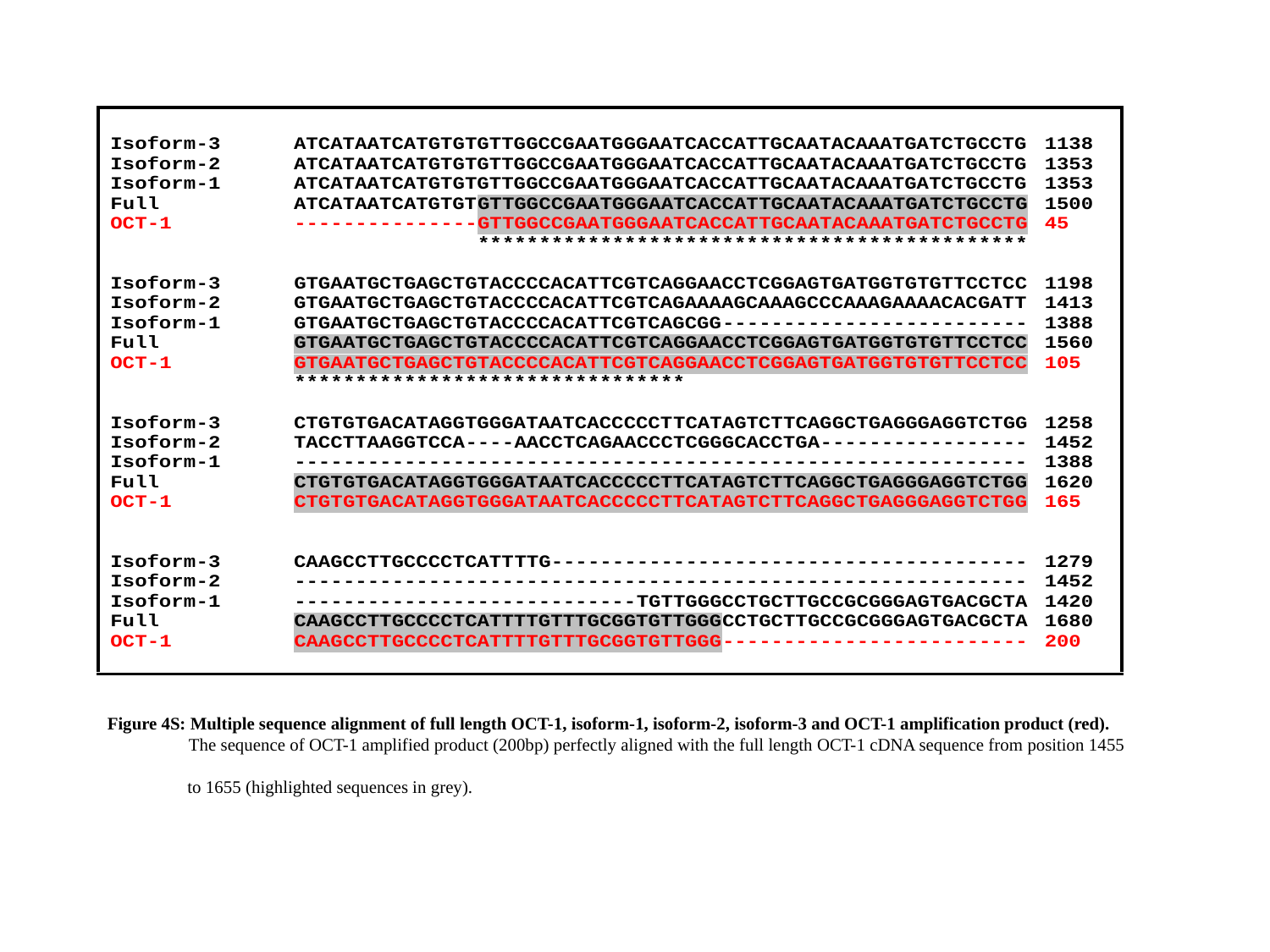

Figure 4S: Multiple sequence alignment of full length OCT-1, isoform-1, isoform-2, isoform-3 and OCT-1 amplification product (red).
 The sequence of OCT-1 amplified product (200bp) perfectly aligned with the full length OCT-1 cDNA sequence from position 1455
 to 1655 (highlighted sequences in grey).

## Slide 5
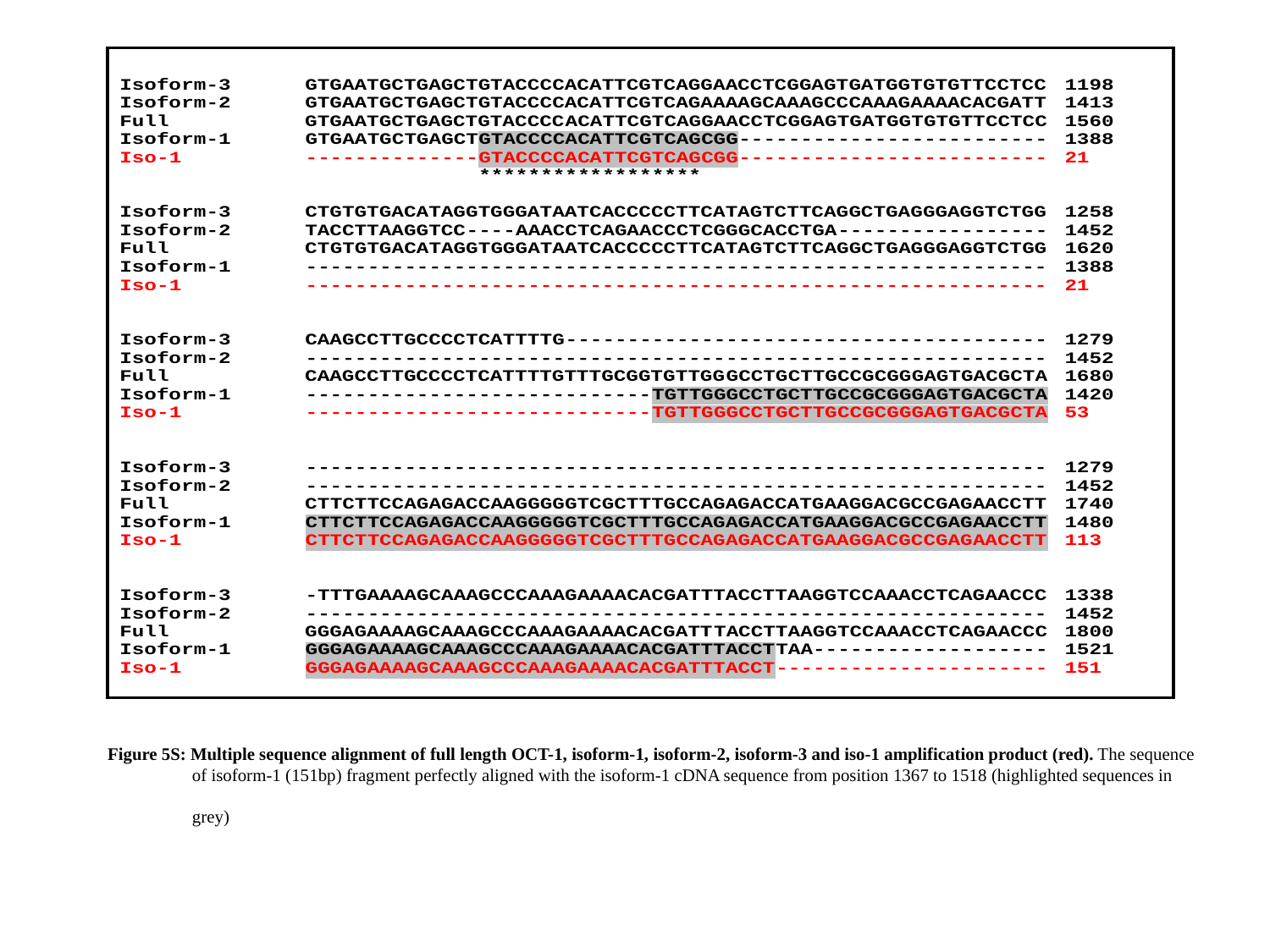

Figure 5S: Multiple sequence alignment of full length OCT-1, isoform-1, isoform-2, isoform-3 and iso-1 amplification product (red). The sequence
 of isoform-1 (151bp) fragment perfectly aligned with the isoform-1 cDNA sequence from position 1367 to 1518 (highlighted sequences in
 grey)

## Slide 6
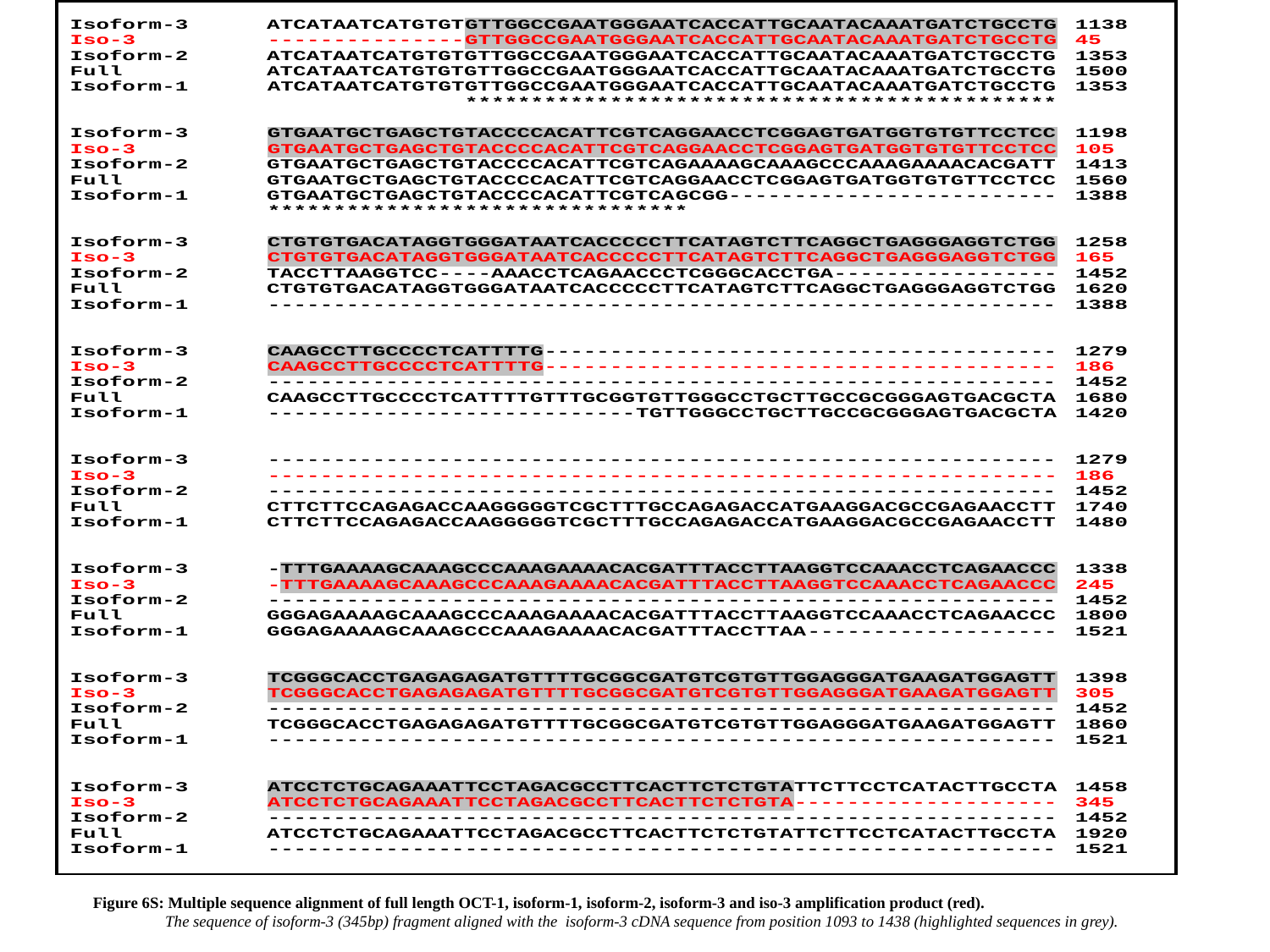

Figure 6S: Multiple sequence alignment of full length OCT-1, isoform-1, isoform-2, isoform-3 and iso-3 amplification product (red).
 The sequence of isoform-3 (345bp) fragment aligned with the isoform-3 cDNA sequence from position 1093 to 1438 (highlighted sequences in grey).

## Slide 7
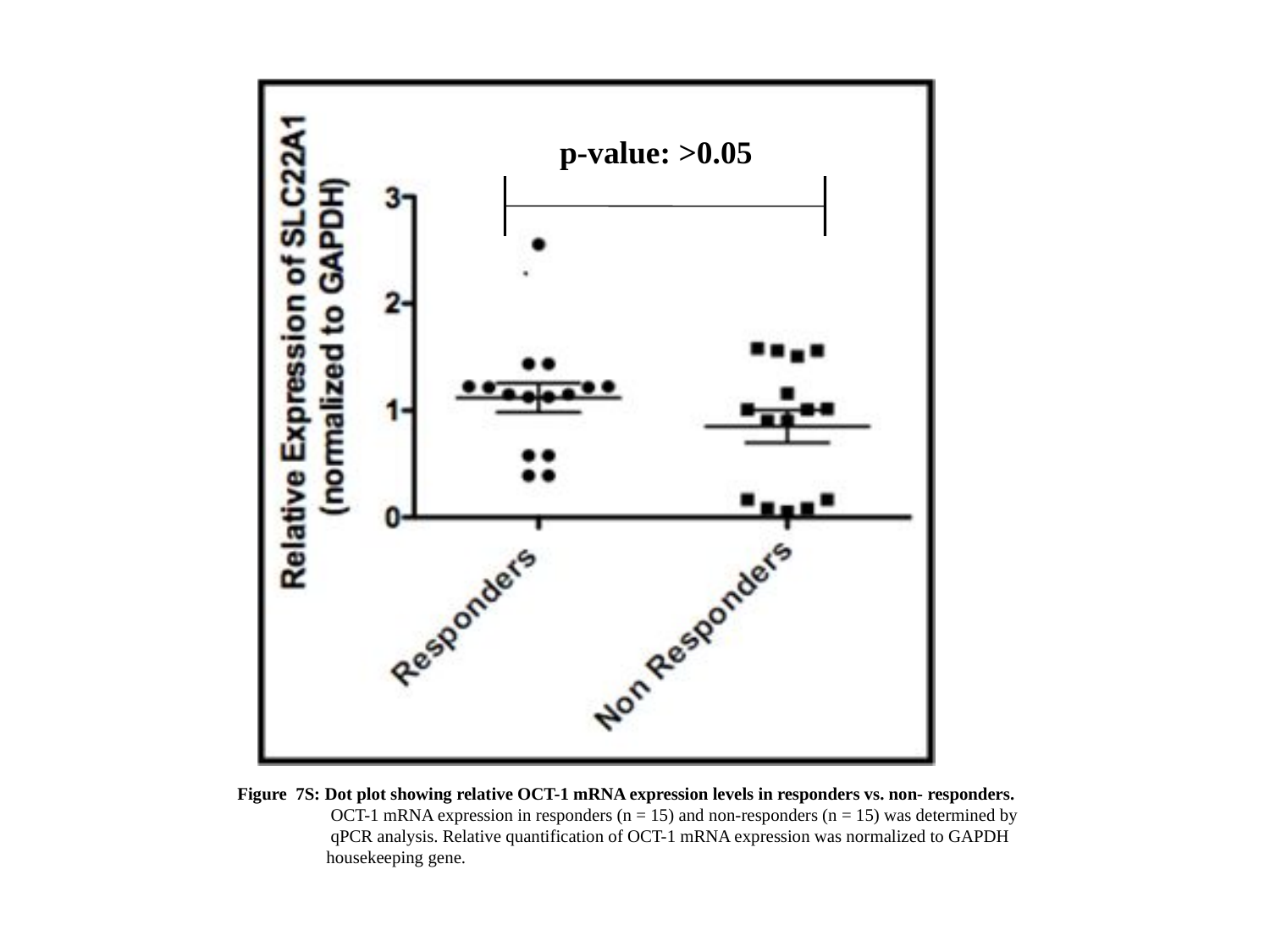

p-value: >0.05
Figure 7S: Dot plot showing relative OCT-1 mRNA expression levels in responders vs. non- responders.
 OCT-1 mRNA expression in responders (n = 15) and non-responders (n = 15) was determined by
 qPCR analysis. Relative quantification of OCT-1 mRNA expression was normalized to GAPDH
 housekeeping gene.

## Slide 8
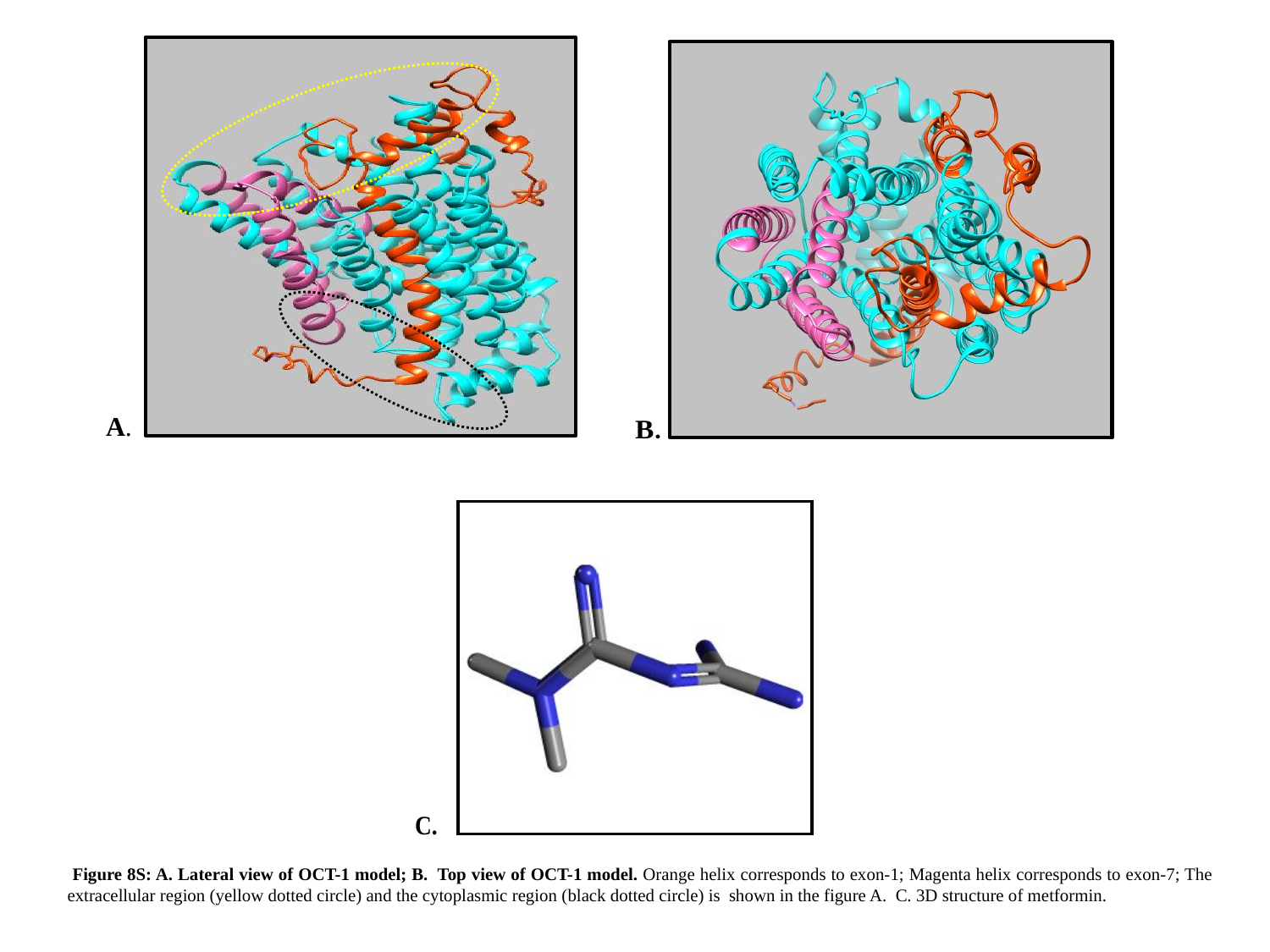

Figure 8S: A. Lateral view of OCT-1 model; B. Top view of OCT-1 model. Orange helix corresponds to exon-1; Magenta helix corresponds to exon-7; The extracellular region (yellow dotted circle) and the cytoplasmic region (black dotted circle) is shown in the figure A. C. 3D structure of metformin.

## Slide 9
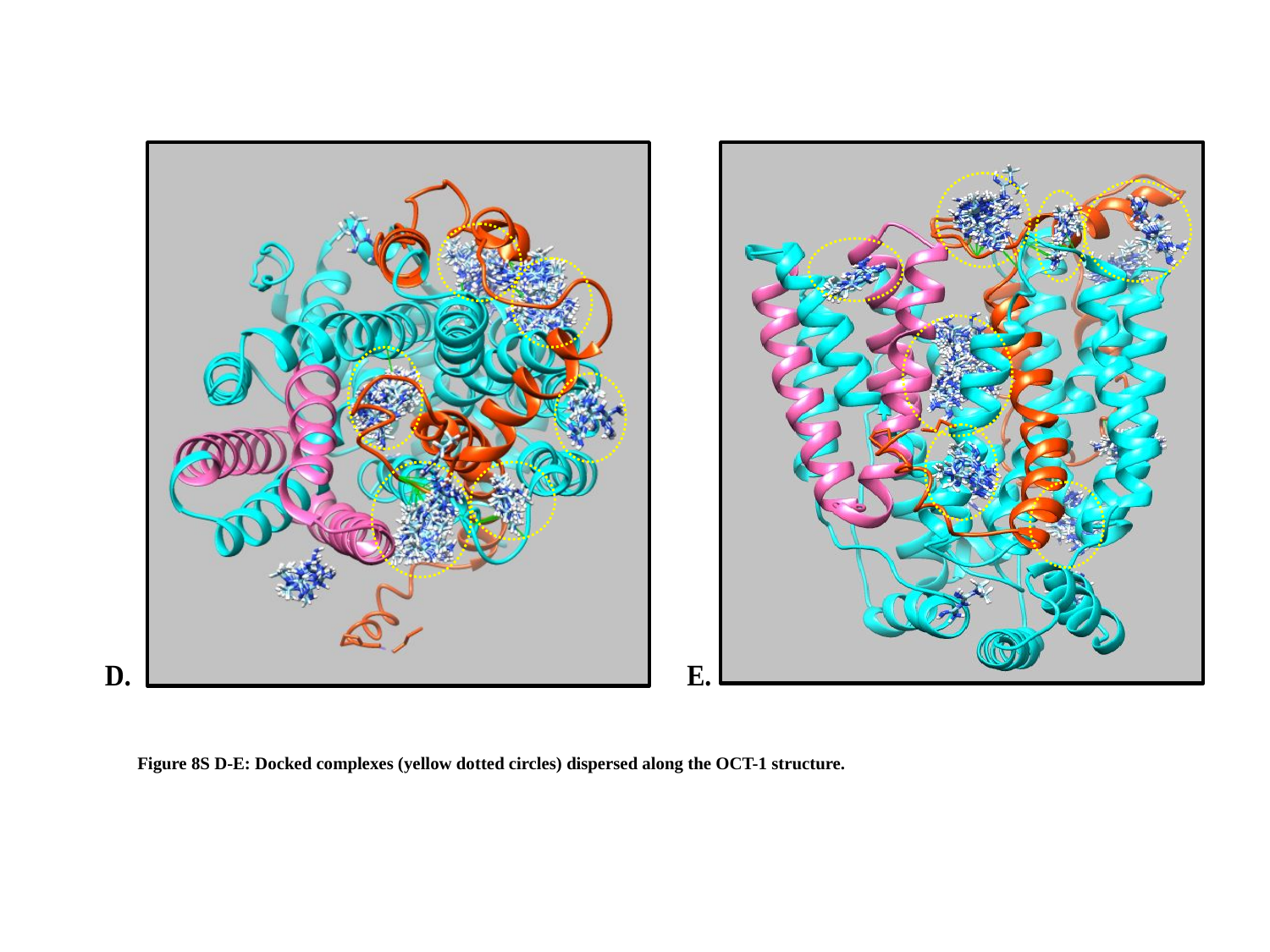

Figure 8S D-E: Docked complexes (yellow dotted circles) dispersed along the OCT-1 structure.

## Slide 10
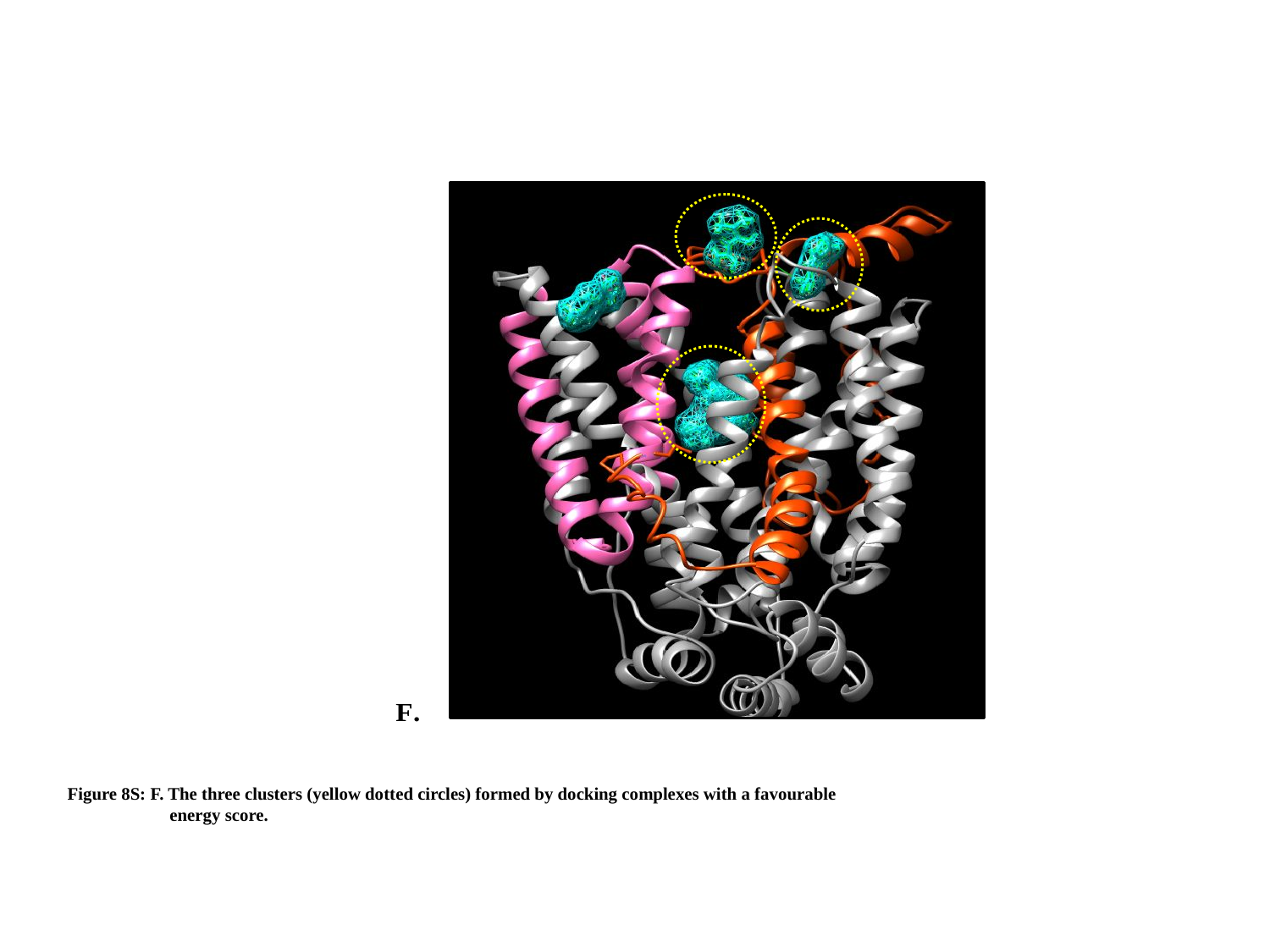

Figure 8S: F. The three clusters (yellow dotted circles) formed by docking complexes with a favourable
 energy score.

## Slide 11
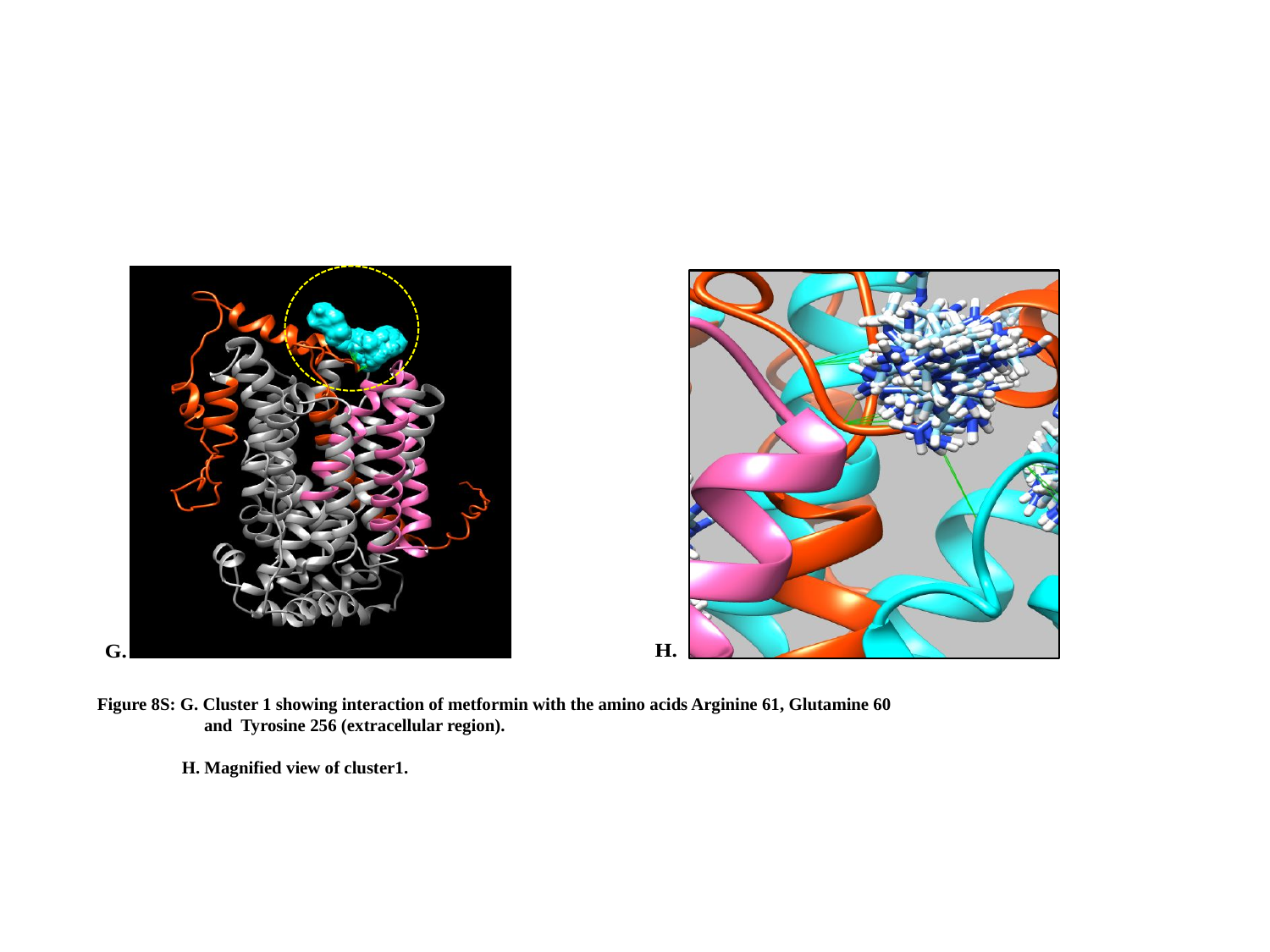

Figure 8S: G. Cluster 1 showing interaction of metformin with the amino acids Arginine 61, Glutamine 60
 and Tyrosine 256 (extracellular region).
 H. Magnified view of cluster1.

## Slide 12
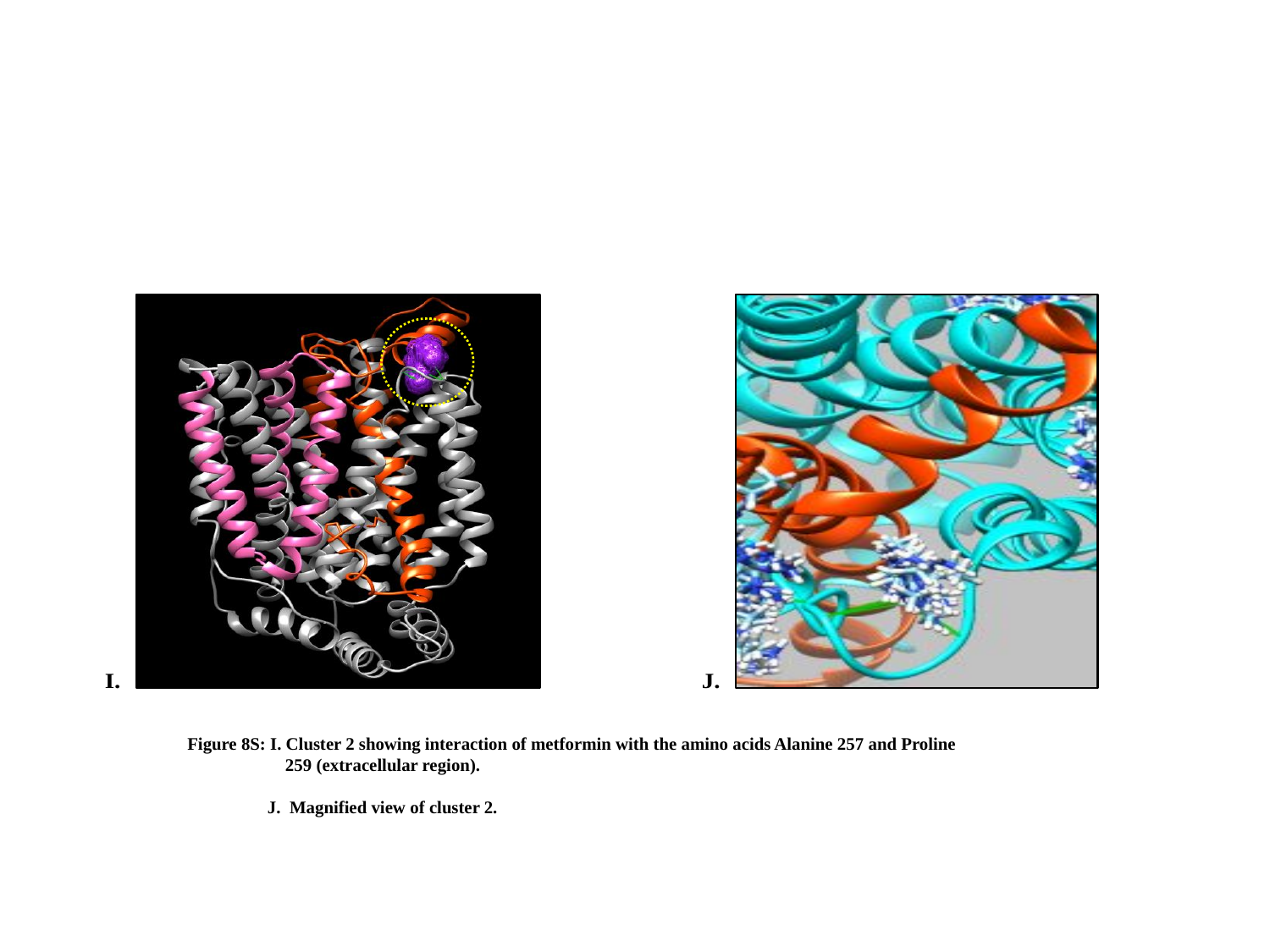

Figure 8S: I. Cluster 2 showing interaction of metformin with the amino acids Alanine 257 and Proline
 259 (extracellular region).
 J. Magnified view of cluster 2.

## Slide 13
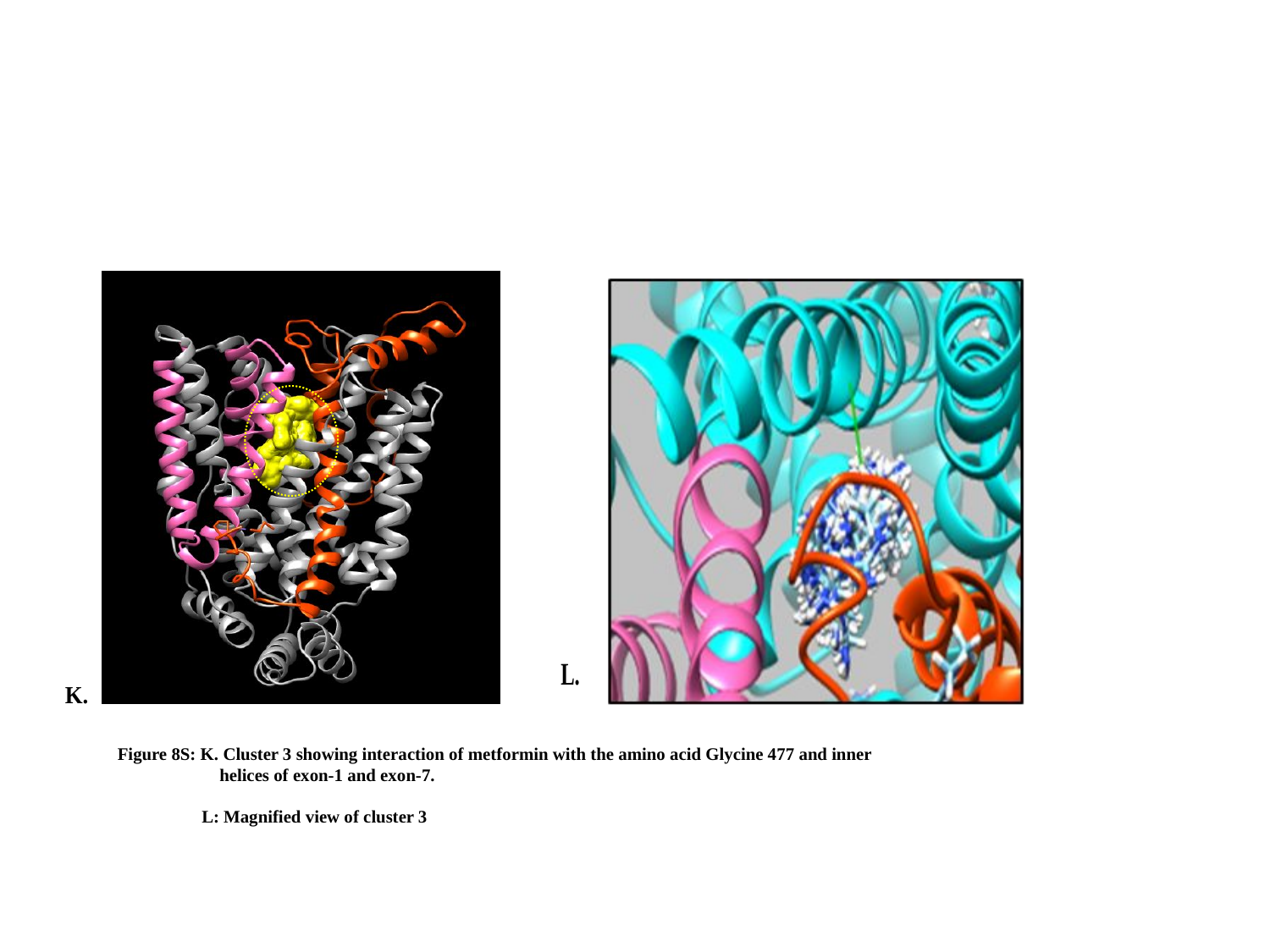

Figure 8S: K. Cluster 3 showing interaction of metformin with the amino acid Glycine 477 and inner
 helices of exon-1 and exon-7.
 L: Magnified view of cluster 3

## Slide 14
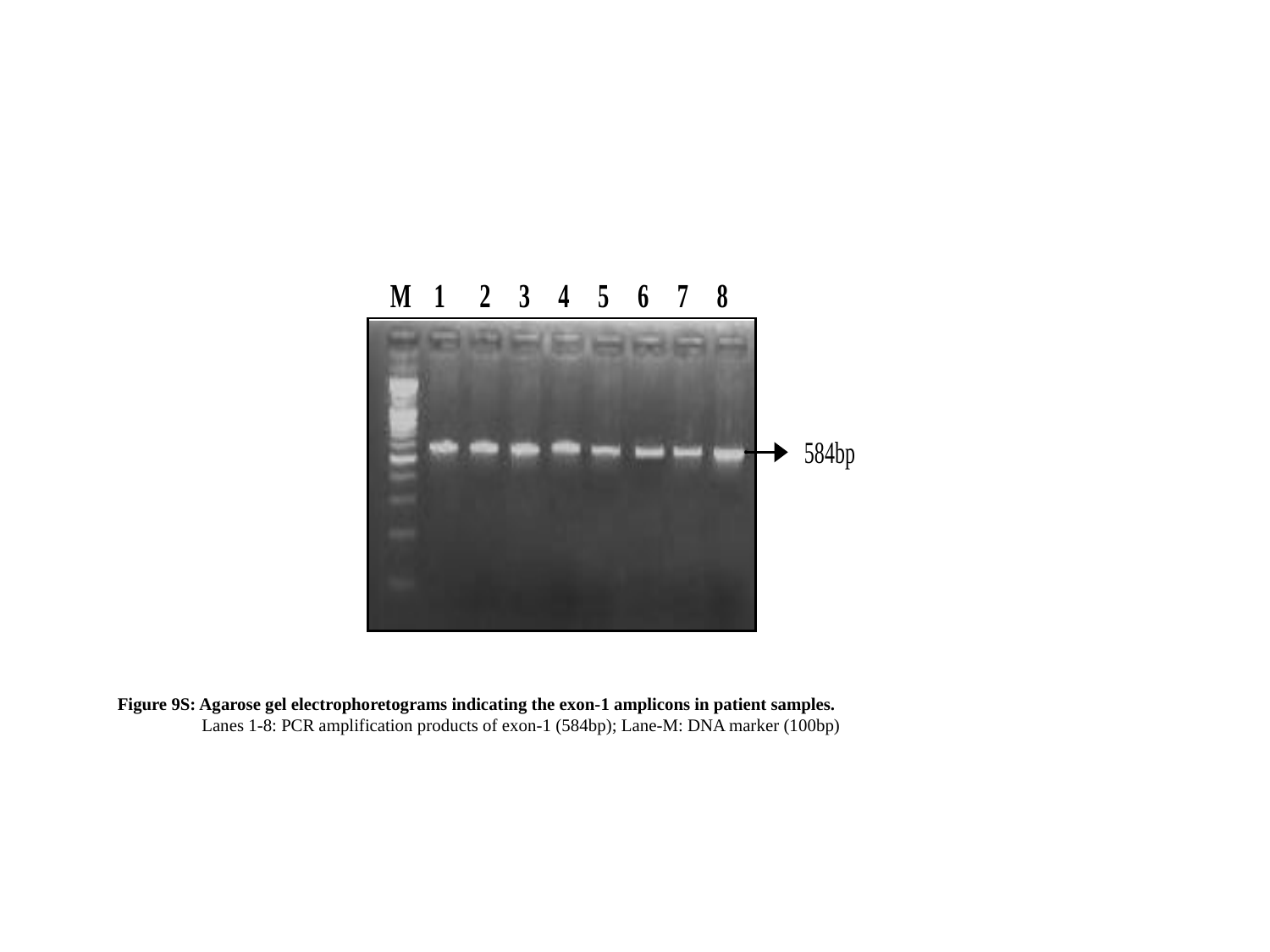

Figure 9S: Agarose gel electrophoretograms indicating the exon-1 amplicons in patient samples.
 Lanes 1-8: PCR amplification products of exon-1 (584bp); Lane-M: DNA marker (100bp)

## Slide 15
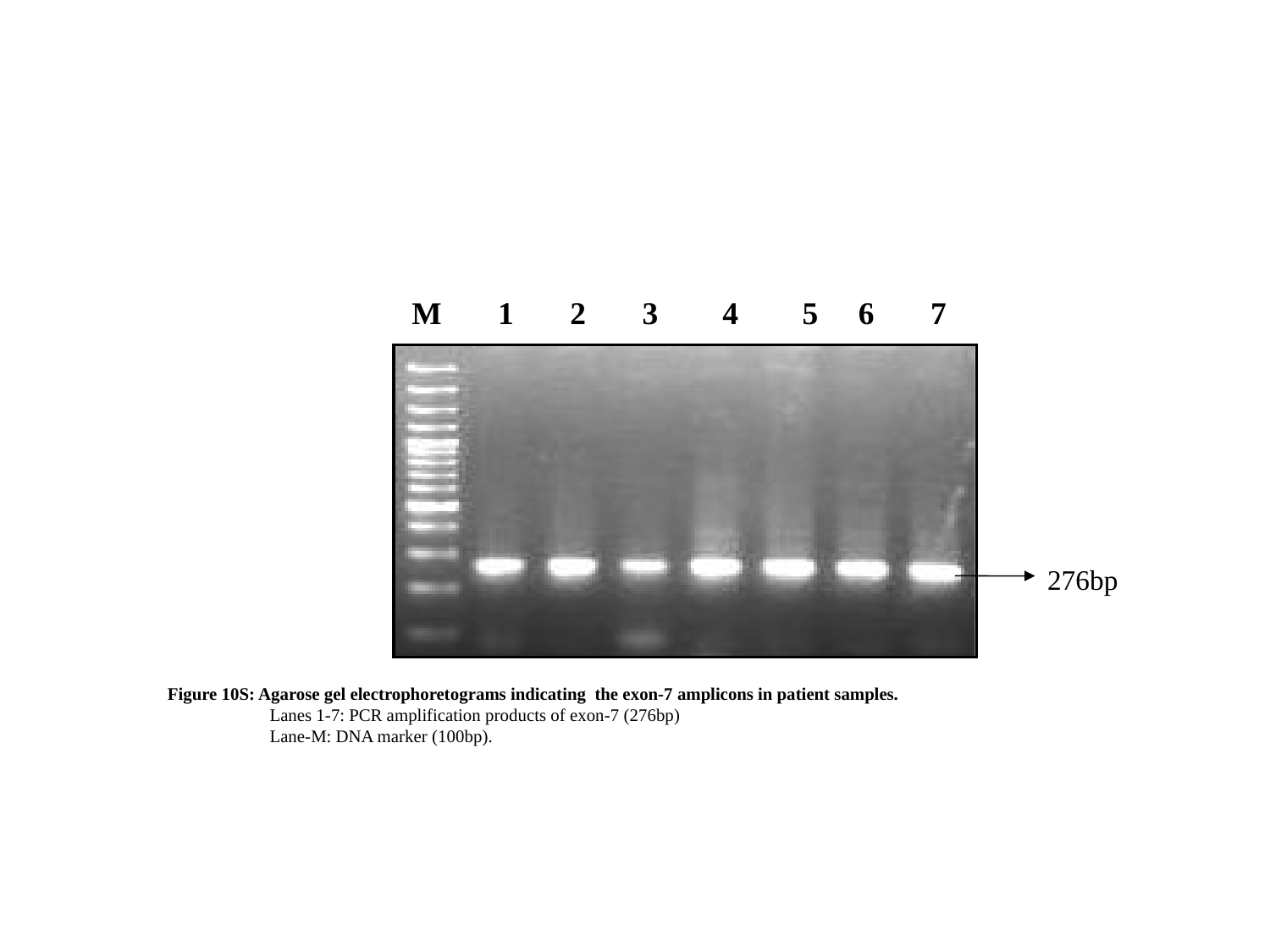

M 1 2 3 4 5 6 7
276bp
Figure 10S: Agarose gel electrophoretograms indicating the exon-7 amplicons in patient samples.
 Lanes 1-7: PCR amplification products of exon-7 (276bp)
 Lane-M: DNA marker (100bp).

## Slide 16
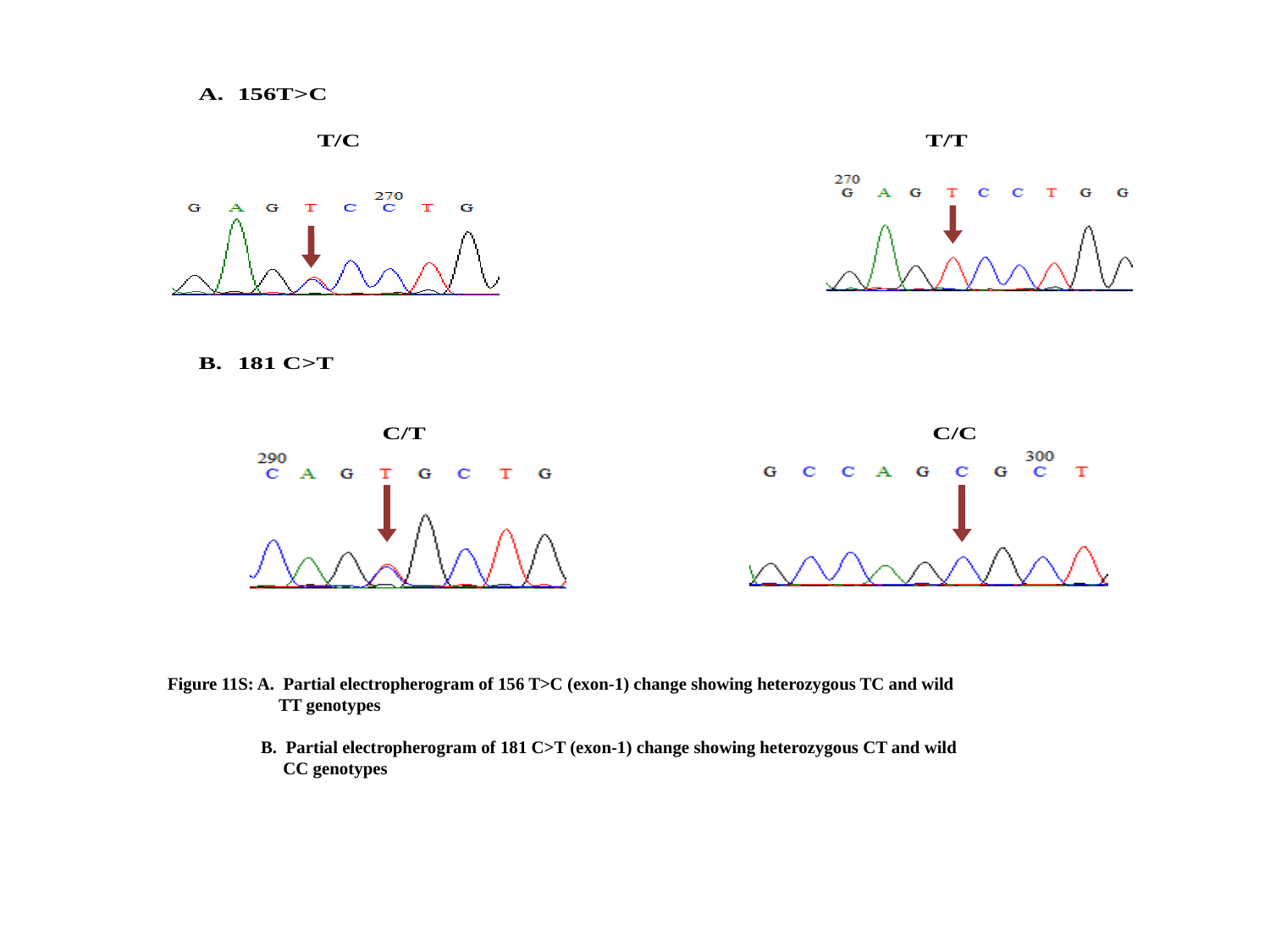

Figure 11S: A. Partial electropherogram of 156 T>C (exon-1) change showing heterozygous TC and wild
 TT genotypes
  B. Partial electropherogram of 181 C>T (exon-1) change showing heterozygous CT and wild
 CC genotypes

## Slide 17
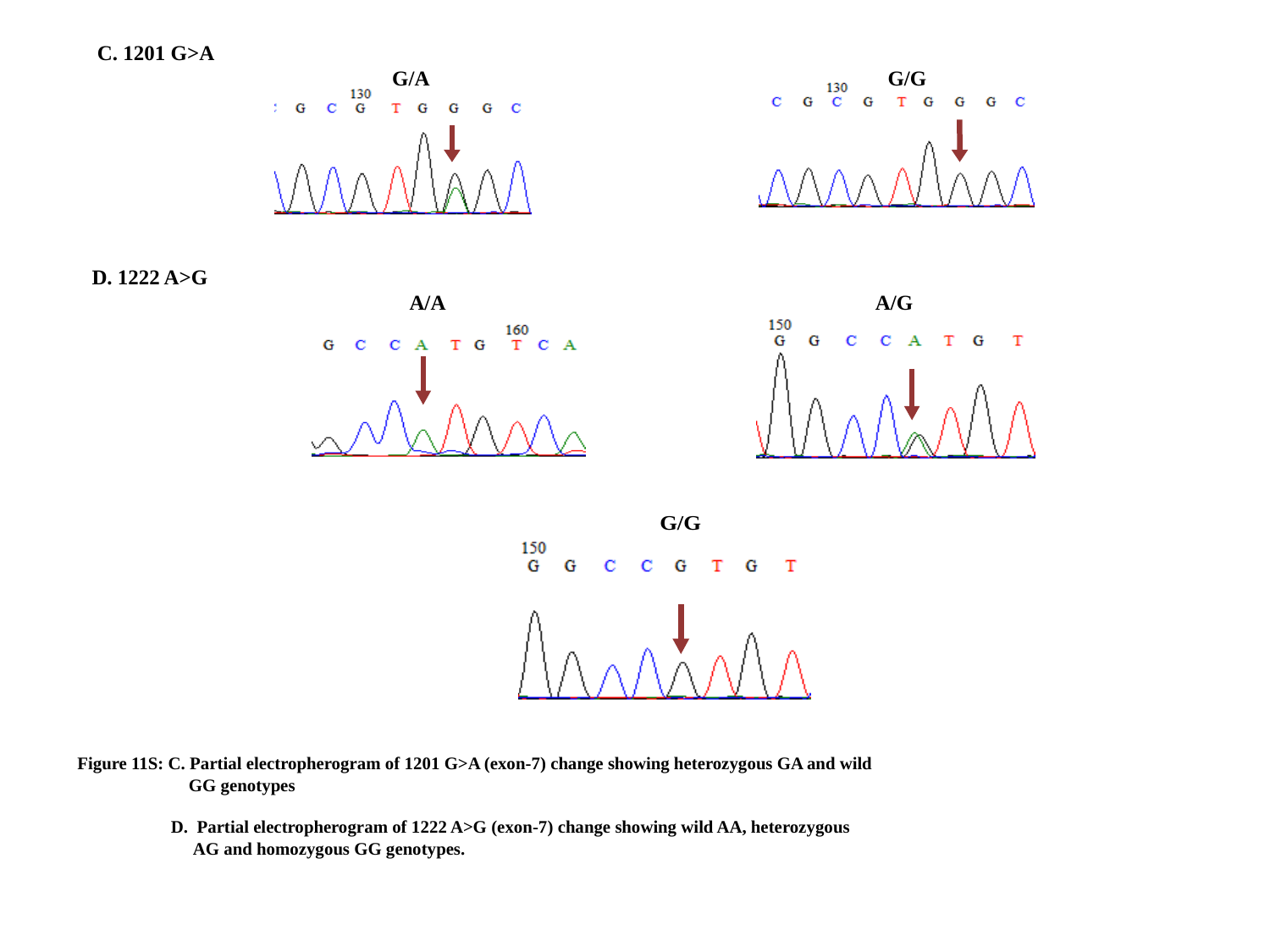

C. 1201 G>A
	 G/A	 G/G
 D. 1222 A>G
 A/A	 A/G
Figure 11S: C. Partial electropherogram of 1201 G>A (exon-7) change showing heterozygous GA and wild
 GG genotypes
 D. Partial electropherogram of 1222 A>G (exon-7) change showing wild AA, heterozygous
 AG and homozygous GG genotypes.

## Slide 18
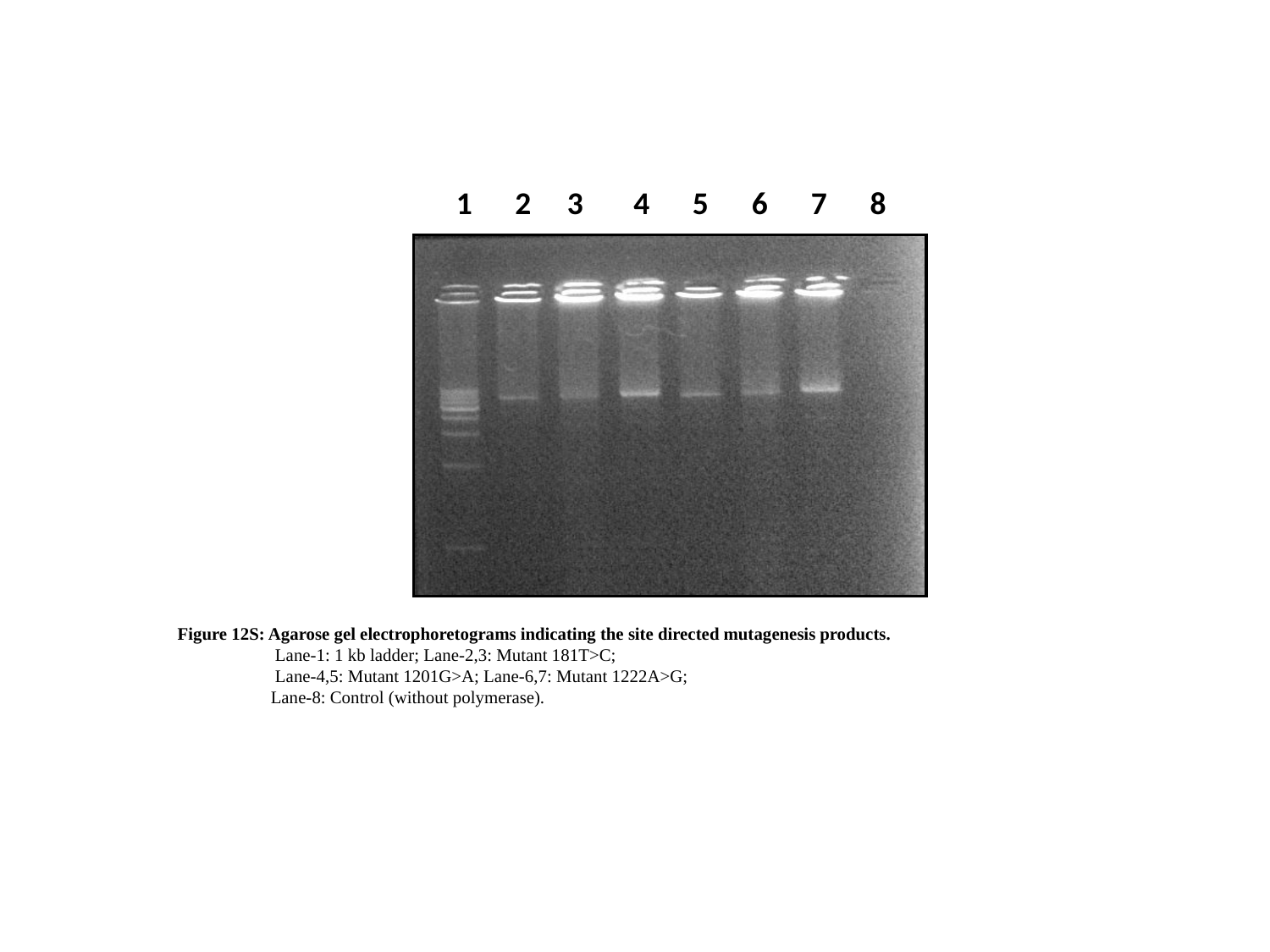

1 2 3 4 5 6 7 8
Figure 12S: Agarose gel electrophoretograms indicating the site directed mutagenesis products.
 Lane-1: 1 kb ladder; Lane-2,3: Mutant 181T>C;
 Lane-4,5: Mutant 1201G>A; Lane-6,7: Mutant 1222A>G;
 Lane-8: Control (without polymerase).
 																																																																																																																																																																																																																																											```````````````````																															`````````````````````````````````````````````````````````````````````````````````````````````````````````````````````````````````````````````````````````````````````````````````````````````````````````````````````````````````````````````````````````````````````````````````````````````````````````````````````````````````````````````````````````````````````````````````````````````````````````````````````````````````````````````````````````````````````````````````````````````````````````````````````````````````````````````````````````````````````````````````````````````````````````````````````````````````````````````````````````````````````````````````````````````````````````````````````````````````````````````````````````````````````````````````````````````````qqqqqqqqqqqqqqqqqqqqqqqqqqqqqqqqqqqqqqqqqqqqqqqqqqqqqqqqqqqqqqqqqqqqqqqqqqqqqqqqqqqqqqqqqqqqqqqqqqqqqqqqqqqqqqqqqqqqqqqqqqqqqqqqqqqqqqqqqqqqqqqqqqqqqqqqqqqqqqqqqqqqqqqqqqqqqqqqqqqqqqqq``````````````qqqqqqqqqqqqqqqqqqqqqqqqqqqqqqqqqqqqqqqqqqqqqqqqqqqqqqqqqqqq`																																																																																	``1```````````````````````1`````````````````````````````````````````````````

## Slide 19
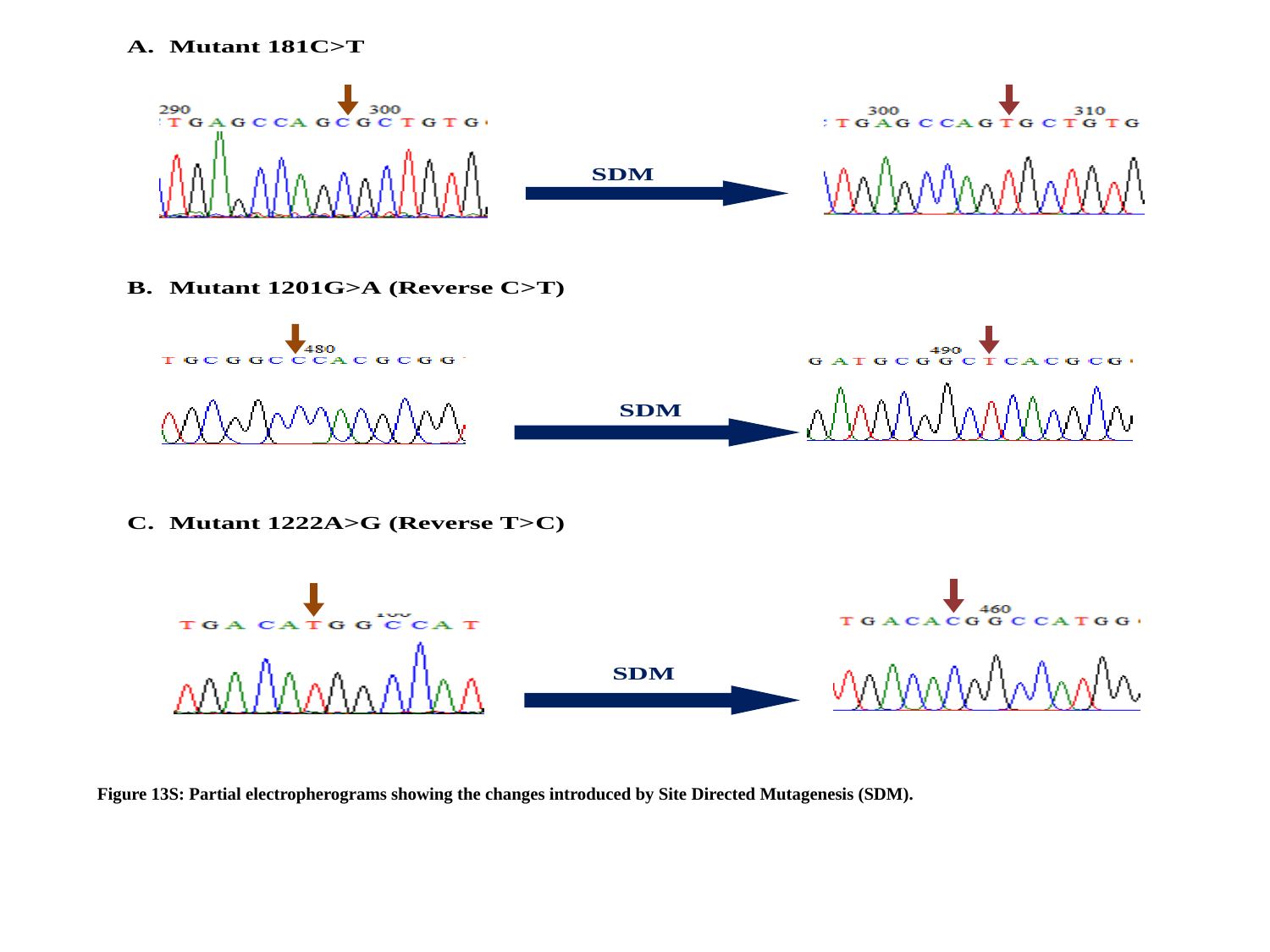

Figure 13S: Partial electropherograms showing the changes introduced by Site Directed Mutagenesis (SDM).

## Slide 20
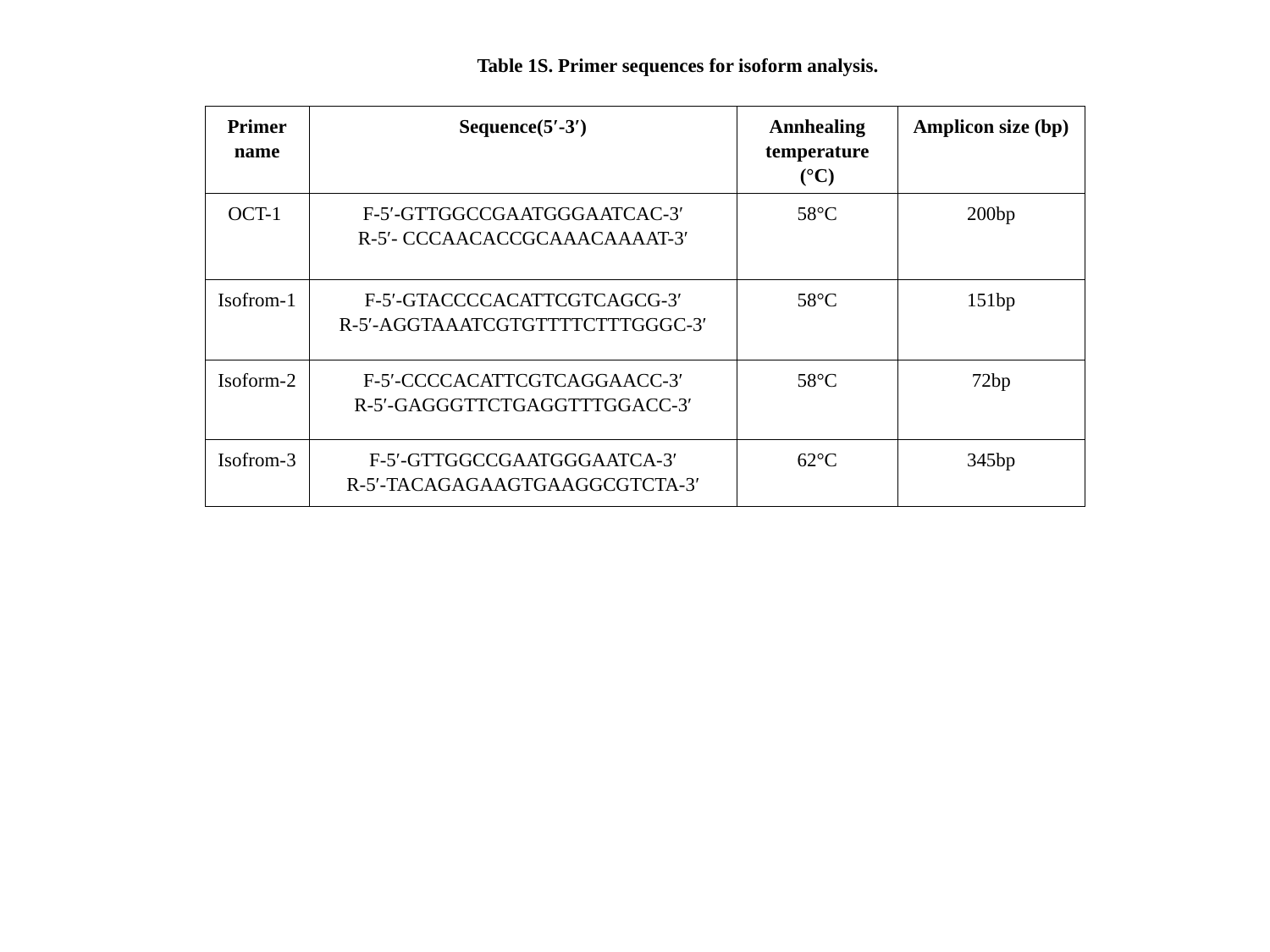

Table 1S. Primer sequences for isoform analysis.
| Primer name | Sequence(5ʹ-3ʹ) | Annhealing temperature (°C) | Amplicon size (bp) |
| --- | --- | --- | --- |
| OCT-1 | F-5ʹ-GTTGGCCGAATGGGAATCAC-3ʹ R-5ʹ- CCCAACACCGCAAACAAAAT-3ʹ | 58°C | 200bp |
| Isofrom-1 | F-5ʹ-GTACCCCACATTCGTCAGCG-3ʹ R-5ʹ-AGGTAAATCGTGTTTTCTTTGGGC-3ʹ | 58°C | 151bp |
| Isoform-2 | F-5ʹ-CCCCACATTCGTCAGGAACC-3ʹ R-5ʹ-GAGGGTTCTGAGGTTTGGACC-3ʹ | 58°C | 72bp |
| Isofrom-3 | F-5ʹ-GTTGGCCGAATGGGAATCA-3ʹ R-5ʹ-TACAGAGAAGTGAAGGCGTCTA-3ʹ | 62°C | 345bp |

## Slide 21
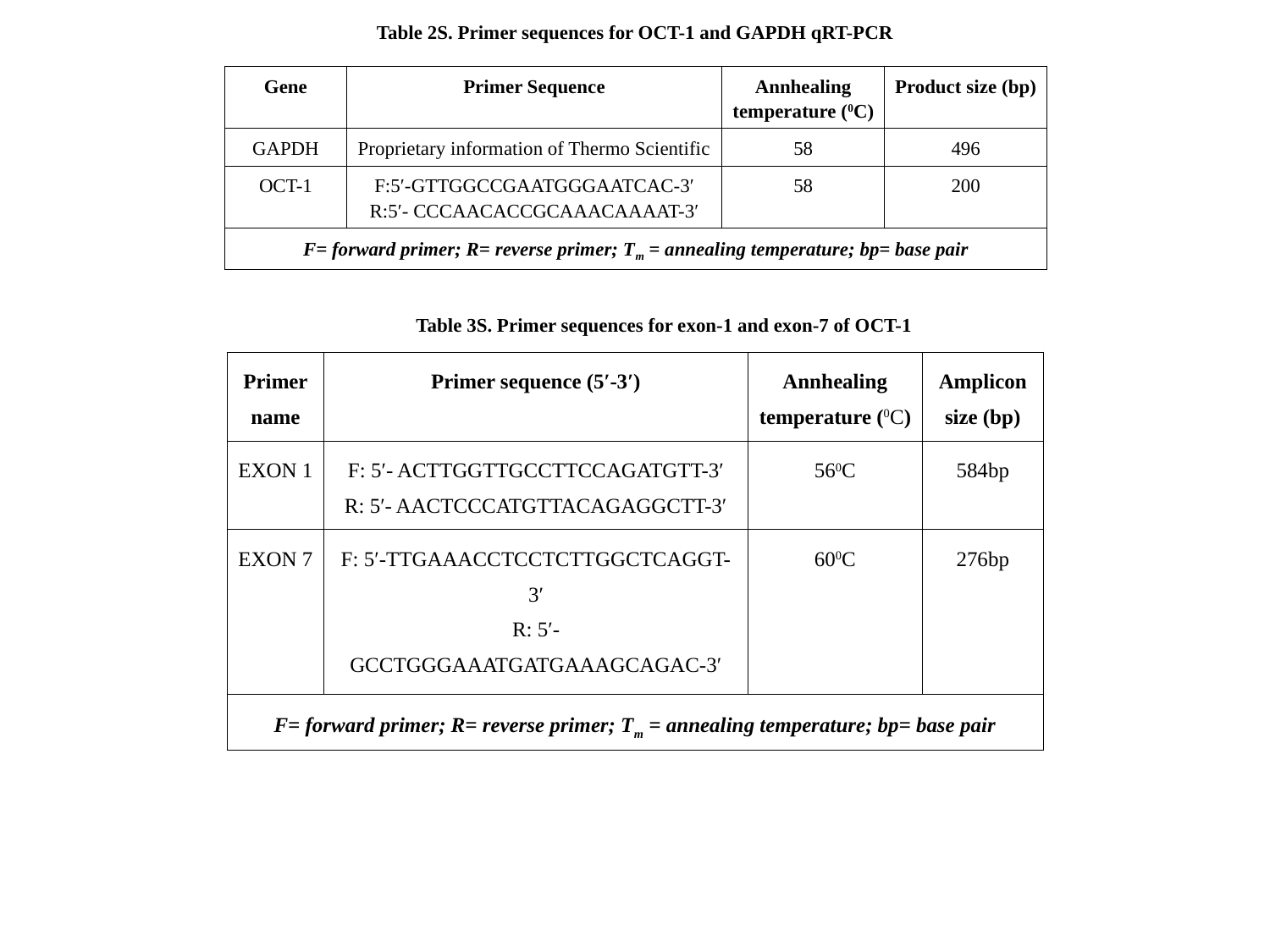

Table 2S. Primer sequences for OCT-1 and GAPDH qRT-PCR
| Gene | Primer Sequence | Annhealing temperature (0C) | Product size (bp) |
| --- | --- | --- | --- |
| GAPDH | Proprietary information of Thermo Scientific | 58 | 496 |
| OCT-1 | F:5ʹ-GTTGGCCGAATGGGAATCAC-3ʹ R:5ʹ- CCCAACACCGCAAACAAAAT-3ʹ | 58 | 200 |
| F= forward primer; R= reverse primer; Tm = annealing temperature; bp= base pair | | | |
Table 3S. Primer sequences for exon-1 and exon-7 of OCT-1
| Primer name | Primer sequence (5ʹ-3ʹ) | Annhealing temperature (0C) | Amplicon size (bp) |
| --- | --- | --- | --- |
| EXON 1 | F: 5ʹ- ACTTGGTTGCCTTCCAGATGTT-3ʹ R: 5ʹ- AACTCCCATGTTACAGAGGCTT-3ʹ | 560C | 584bp |
| EXON 7 | F: 5ʹ-TTGAAACCTCCTCTTGGCTCAGGT-3ʹ R: 5ʹ- GCCTGGGAAATGATGAAAGCAGAC-3ʹ | 600C | 276bp |
| F= forward primer; R= reverse primer; Tm = annealing temperature; bp= base pair | | | |

## Slide 22
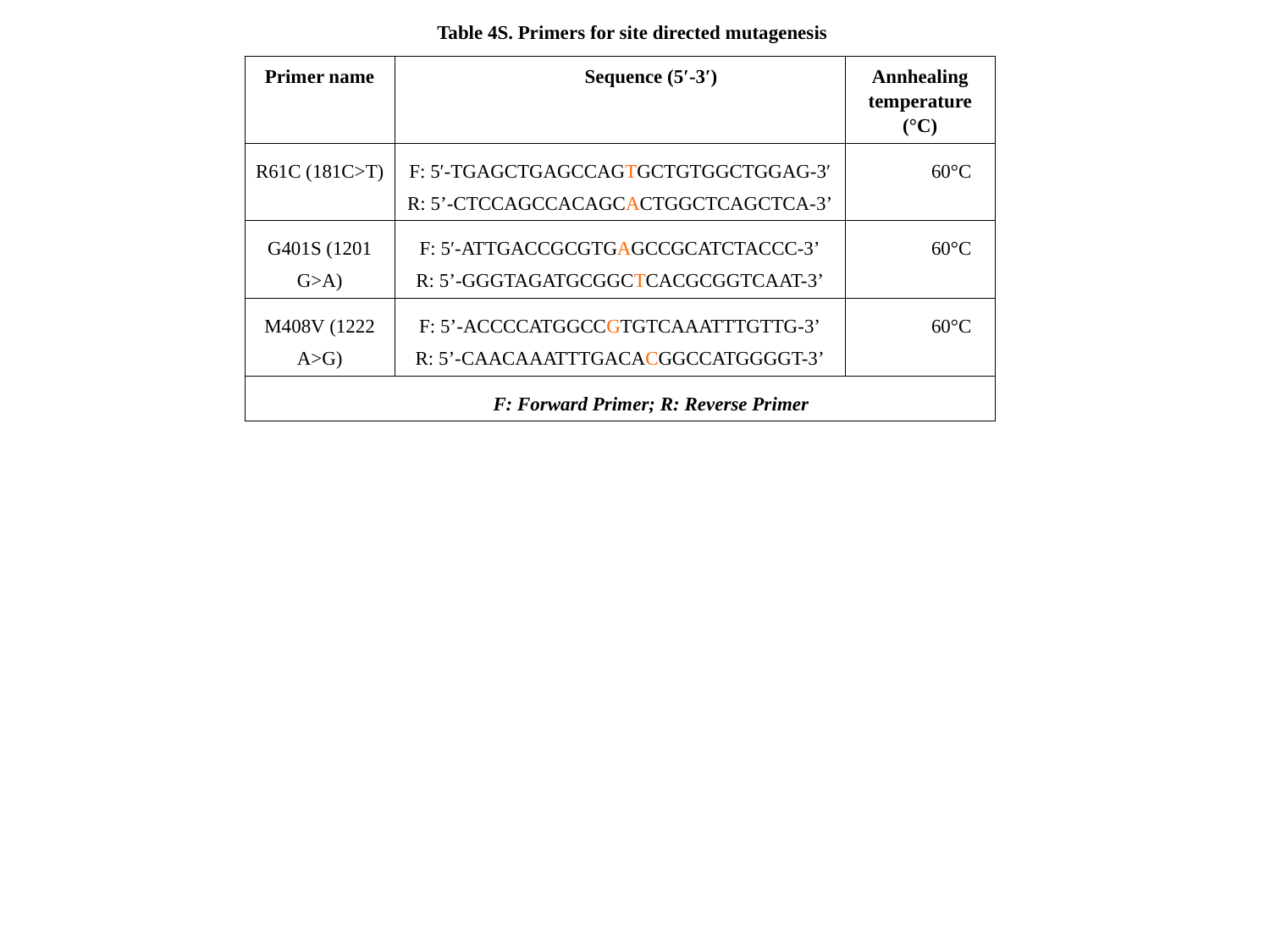

Table 4S. Primers for site directed mutagenesis
| Primer name | Sequence (5ʹ-3ʹ) | Annhealing temperature (°C) |
| --- | --- | --- |
| R61C (181C>T) | F: 5ʹ-TGAGCTGAGCCAGTGCTGTGGCTGGAG-3ʹ R: 5’-CTCCAGCCACAGCACTGGCTCAGCTCA-3’ | 60°C |
| G401S (1201 G>A) | F: 5ʹ-ATTGACCGCGTGAGCCGCATCTACCC-3’ R: 5’-GGGTAGATGCGGCTCACGCGGTCAAT-3’ | 60°C |
| M408V (1222 A>G) | F: 5’-ACCCCATGGCCGTGTCAAATTTGTTG-3’ R: 5’-CAACAAATTTGACACGGCCATGGGGT-3’ | 60°C |
| F: Forward Primer; R: Reverse Primer | | |

## Slide 23
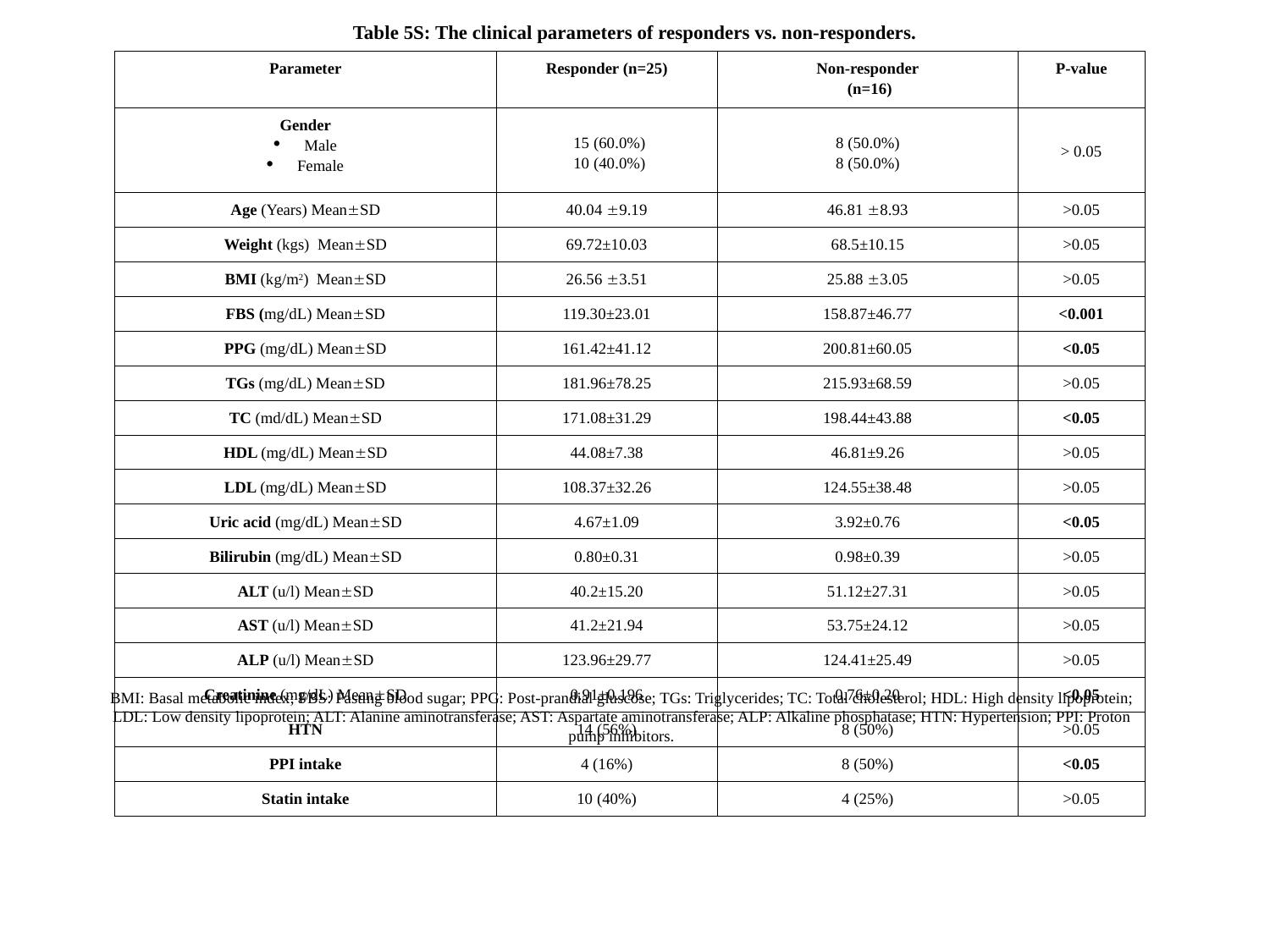

Table 5S: The clinical parameters of responders vs. non-responders.
| Parameter | Responder (n=25) | Non-responder (n=16) | P-value |
| --- | --- | --- | --- |
| Gender Male Female | 15 (60.0%) 10 (40.0%) | 8 (50.0%) 8 (50.0%) | > 0.05 |
| Age (Years) Mean±SD | 40.04 ±9.19 | 46.81 ±8.93 | >0.05 |
| Weight (kgs) Mean±SD | 69.72±10.03 | 68.5±10.15 | >0.05 |
| BMI (kg/m2) Mean±SD | 26.56 ±3.51 | 25.88 ±3.05 | >0.05 |
| FBS (mg/dL) Mean±SD | 119.30±23.01 | 158.87±46.77 | <0.001 |
| PPG (mg/dL) Mean±SD | 161.42±41.12 | 200.81±60.05 | <0.05 |
| TGs (mg/dL) Mean±SD | 181.96±78.25 | 215.93±68.59 | >0.05 |
| TC (md/dL) Mean±SD | 171.08±31.29 | 198.44±43.88 | <0.05 |
| HDL (mg/dL) Mean±SD | 44.08±7.38 | 46.81±9.26 | >0.05 |
| LDL (mg/dL) Mean±SD | 108.37±32.26 | 124.55±38.48 | >0.05 |
| Uric acid (mg/dL) Mean±SD | 4.67±1.09 | 3.92±0.76 | <0.05 |
| Bilirubin (mg/dL) Mean±SD | 0.80±0.31 | 0.98±0.39 | >0.05 |
| ALT (u/l) Mean±SD | 40.2±15.20 | 51.12±27.31 | >0.05 |
| AST (u/l) Mean±SD | 41.2±21.94 | 53.75±24.12 | >0.05 |
| ALP (u/l) Mean±SD | 123.96±29.77 | 124.41±25.49 | >0.05 |
| Creatinine (mg/dL) Mean±SD | 0.91±0.196 | 0.76±0.20 | <0.05 |
| HTN | 14 (56%) | 8 (50%) | >0.05 |
| PPI intake | 4 (16%) | 8 (50%) | <0.05 |
| Statin intake | 10 (40%) | 4 (25%) | >0.05 |
BMI: Basal metabolic index; FBS: Fasting blood sugar; PPG: Post-prandial gluscose; TGs: Triglycerides; TC: Total cholesterol; HDL: High density lipoprotein; LDL: Low density lipoprotein; ALT: Alanine aminotransferase; AST: Aspartate aminotransferase; ALP: Alkaline phosphatase; HTN: Hypertension; PPI: Proton pump inhibitors.

## Slide 24
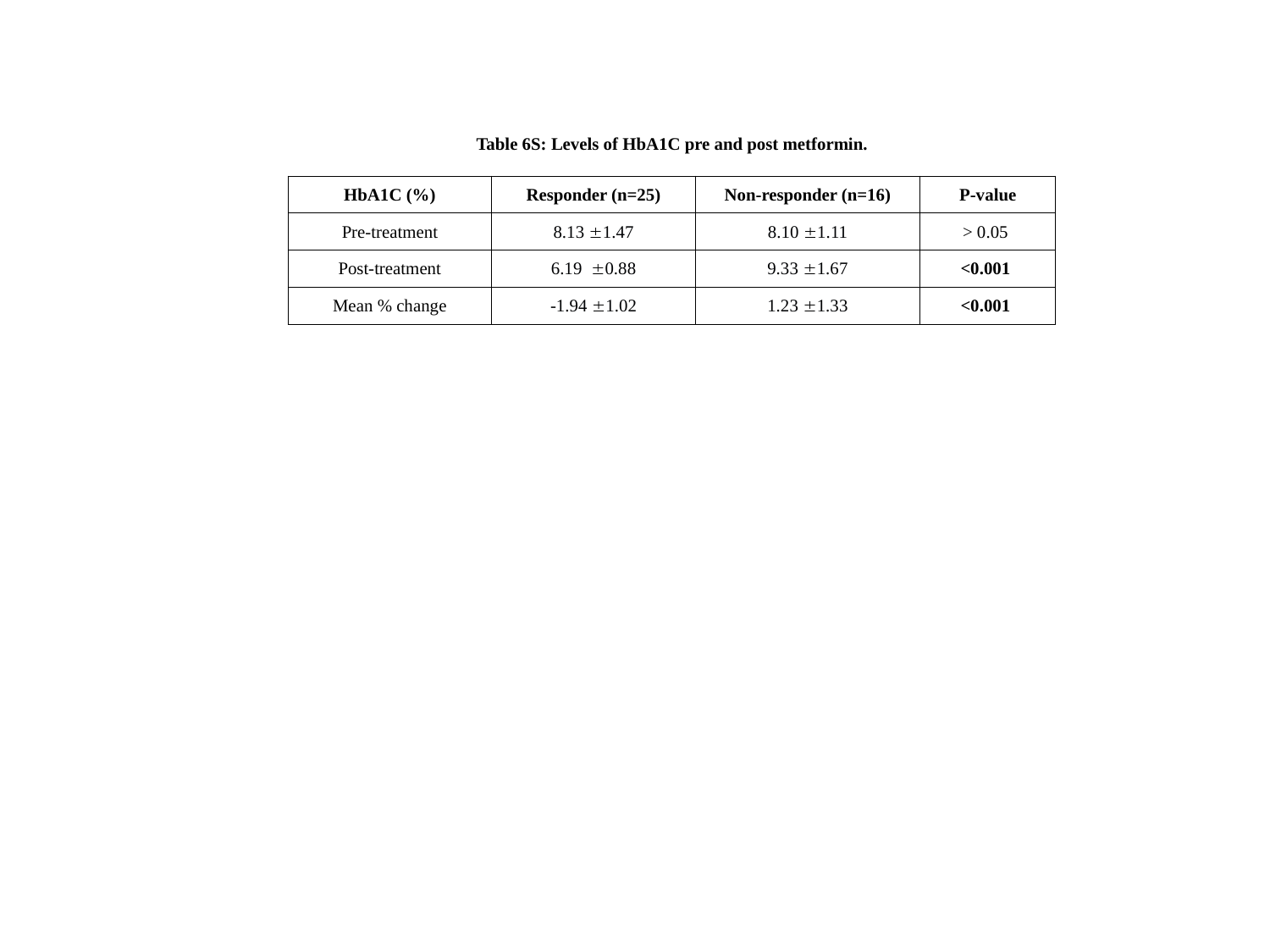

Table 6S: Levels of HbA1C pre and post metformin.
| HbA1C (%) | Responder (n=25) | Non-responder (n=16) | P-value |
| --- | --- | --- | --- |
| Pre-treatment | 8.13 ±1.47 | 8.10 ±1.11 | > 0.05 |
| Post-treatment | 6.19 ±0.88 | 9.33 ±1.67 | <0.001 |
| Mean % change | -1.94 ±1.02 | 1.23 ±1.33 | <0.001 |

## Slide 25
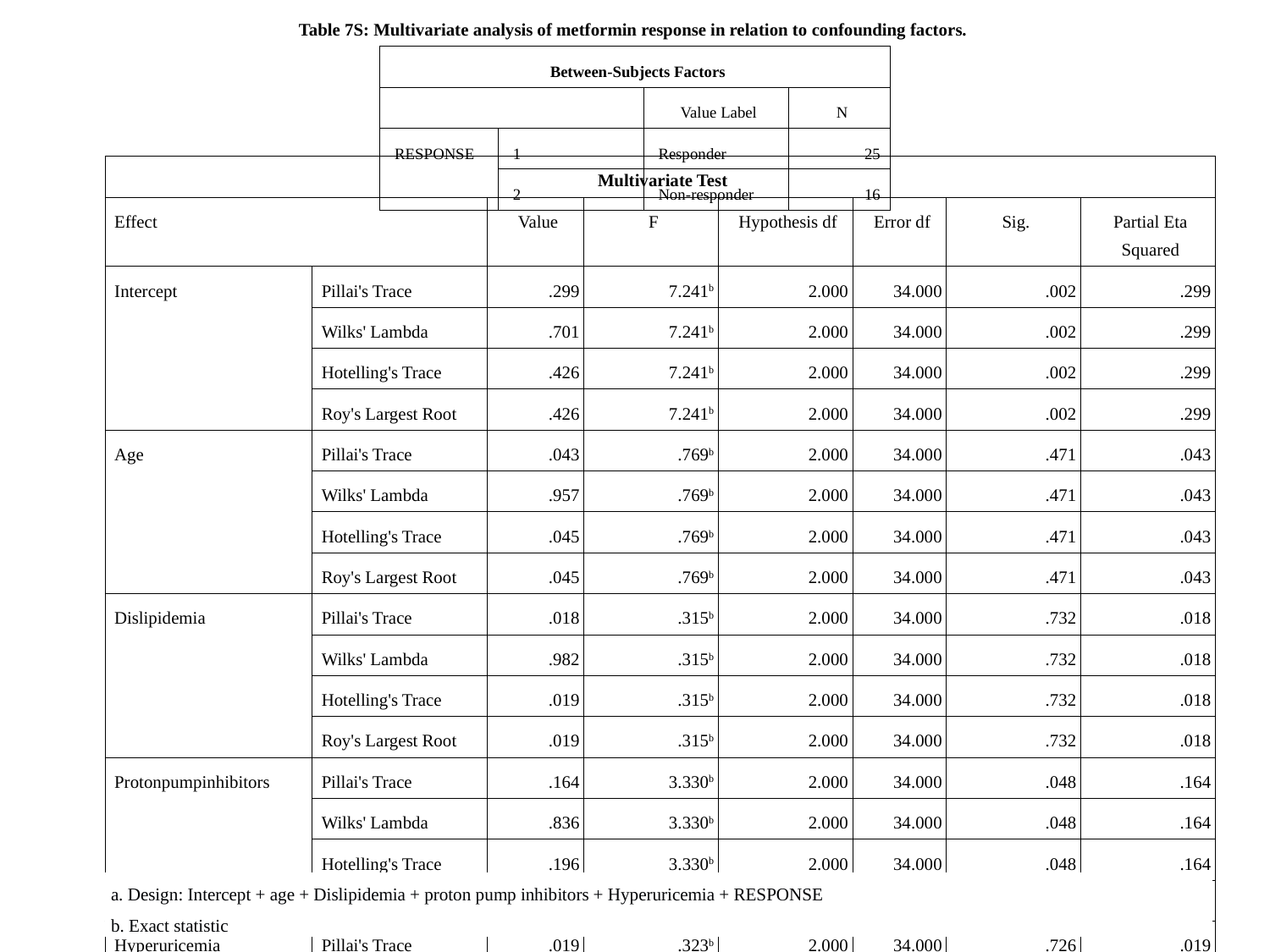

Table 7S: Multivariate analysis of metformin response in relation to confounding factors.
| Between-Subjects Factors | | | |
| --- | --- | --- | --- |
| | | Value Label | N |
| RESPONSE | 1 | Responder | 25 |
| | 2 | Non-responder | 16 |
| Multivariate Test | | | | | | | |
| --- | --- | --- | --- | --- | --- | --- | --- |
| Effect | | Value | F | Hypothesis df | Error df | Sig. | Partial Eta Squared |
| Intercept | Pillai's Trace | .299 | 7.241b | 2.000 | 34.000 | .002 | .299 |
| | Wilks' Lambda | .701 | 7.241b | 2.000 | 34.000 | .002 | .299 |
| | Hotelling's Trace | .426 | 7.241b | 2.000 | 34.000 | .002 | .299 |
| | Roy's Largest Root | .426 | 7.241b | 2.000 | 34.000 | .002 | .299 |
| Age | Pillai's Trace | .043 | .769b | 2.000 | 34.000 | .471 | .043 |
| | Wilks' Lambda | .957 | .769b | 2.000 | 34.000 | .471 | .043 |
| | Hotelling's Trace | .045 | .769b | 2.000 | 34.000 | .471 | .043 |
| | Roy's Largest Root | .045 | .769b | 2.000 | 34.000 | .471 | .043 |
| Dislipidemia | Pillai's Trace | .018 | .315b | 2.000 | 34.000 | .732 | .018 |
| | Wilks' Lambda | .982 | .315b | 2.000 | 34.000 | .732 | .018 |
| | Hotelling's Trace | .019 | .315b | 2.000 | 34.000 | .732 | .018 |
| | Roy's Largest Root | .019 | .315b | 2.000 | 34.000 | .732 | .018 |
| Protonpumpinhibitors | Pillai's Trace | .164 | 3.330b | 2.000 | 34.000 | .048 | .164 |
| | Wilks' Lambda | .836 | 3.330b | 2.000 | 34.000 | .048 | .164 |
| | Hotelling's Trace | .196 | 3.330b | 2.000 | 34.000 | .048 | .164 |
| | Roy's Largest Root | .196 | 3.330b | 2.000 | 34.000 | .048 | .164 |
| Hyperuricemia | Pillai's Trace | .019 | .323b | 2.000 | 34.000 | .726 | .019 |
| | Wilks' Lambda | .981 | .323b | 2.000 | 34.000 | .726 | .019 |
| | Hotelling's Trace | .019 | .323b | 2.000 | 34.000 | .726 | .019 |
| | Roy's Largest Root | .019 | .323b | 2.000 | 34.000 | .726 | .019 |
| RESPONSE | Pillai's Trace | .475 | 15.355b | 2.000 | 34.000 | .000 | .475 |
| | Wilks' Lambda | .525 | 15.355b | 2.000 | 34.000 | .000 | .475 |
| | Hotelling's Trace | .903 | 15.355b | 2.000 | 34.000 | .000 | .475 |
| | Roy's Largest Root | .903 | 15.355b | 2.000 | 34.000 | .000 | .475 |

## Slide 26
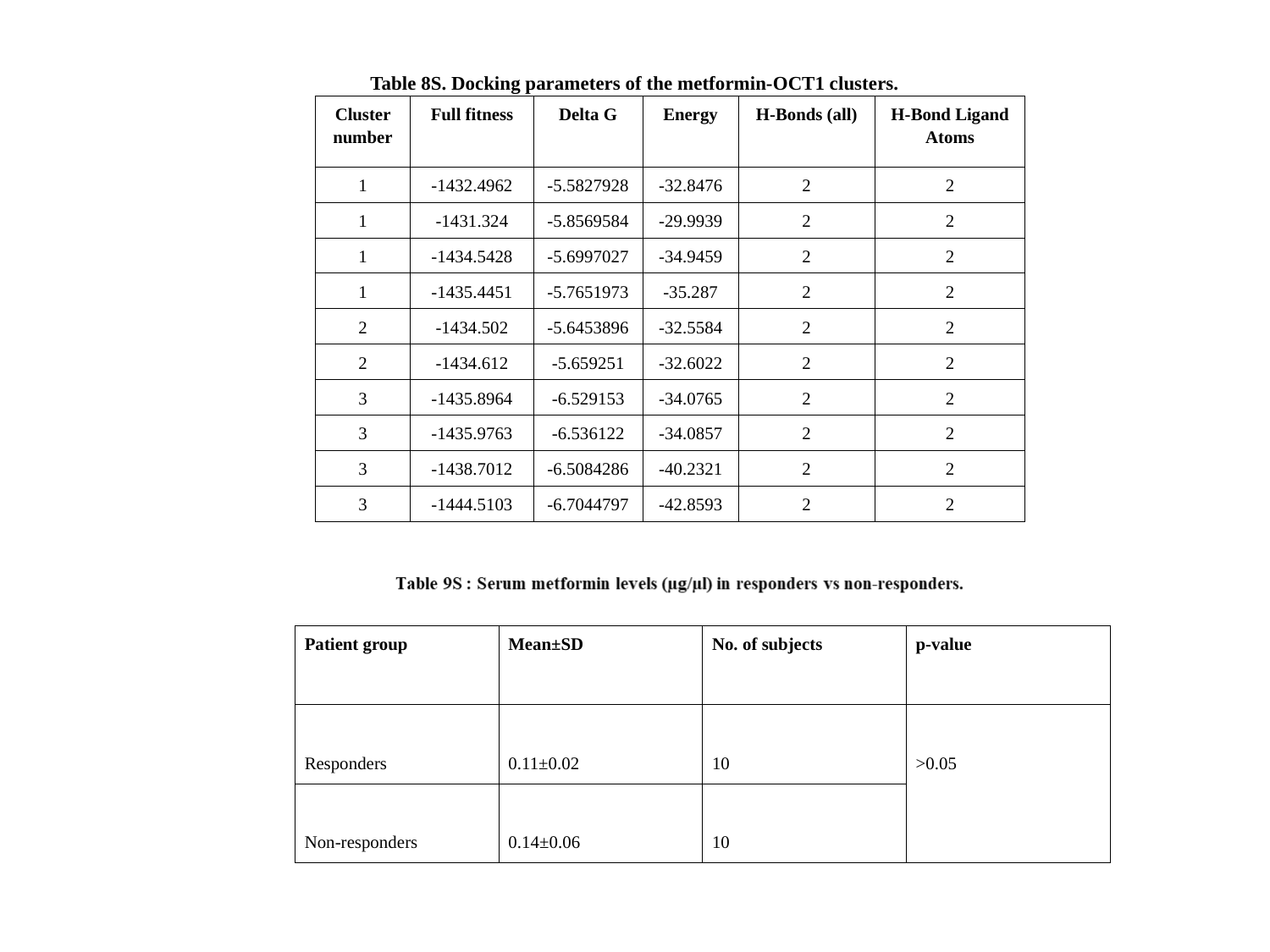

Table 8S. Docking parameters of the metformin-OCT1 clusters.
| Cluster number | Full fitness | Delta G | Energy | H-Bonds (all) | H-Bond Ligand Atoms |
| --- | --- | --- | --- | --- | --- |
| 1 | -1432.4962 | -5.5827928 | -32.8476 | 2 | 2 |
| 1 | -1431.324 | -5.8569584 | -29.9939 | 2 | 2 |
| 1 | -1434.5428 | -5.6997027 | -34.9459 | 2 | 2 |
| 1 | -1435.4451 | -5.7651973 | -35.287 | 2 | 2 |
| 2 | -1434.502 | -5.6453896 | -32.5584 | 2 | 2 |
| 2 | -1434.612 | -5.659251 | -32.6022 | 2 | 2 |
| 3 | -1435.8964 | -6.529153 | -34.0765 | 2 | 2 |
| 3 | -1435.9763 | -6.536122 | -34.0857 | 2 | 2 |
| 3 | -1438.7012 | -6.5084286 | -40.2321 | 2 | 2 |
| 3 | -1444.5103 | -6.7044797 | -42.8593 | 2 | 2 |
| Patient group | Mean±SD | No. of subjects | p-value |
| --- | --- | --- | --- |
| Responders | 0.11±0.02 | 10 | >0.05 |
| Non-responders | 0.14±0.06 | 10 | |
