# Supplementary figures and images for: Role of human organic cation transporter-1 (OCT-1/SLC22A1) in modulating the response to metformin in patients with type 2 diabetes
Source: BMC Endocr Disord. 2022 May 26;22:140. doi: 10.1186/s12902-022-01033-3 (PMC9137212; doi:10.1186/s12902-022-01033-3)

## Slide 1
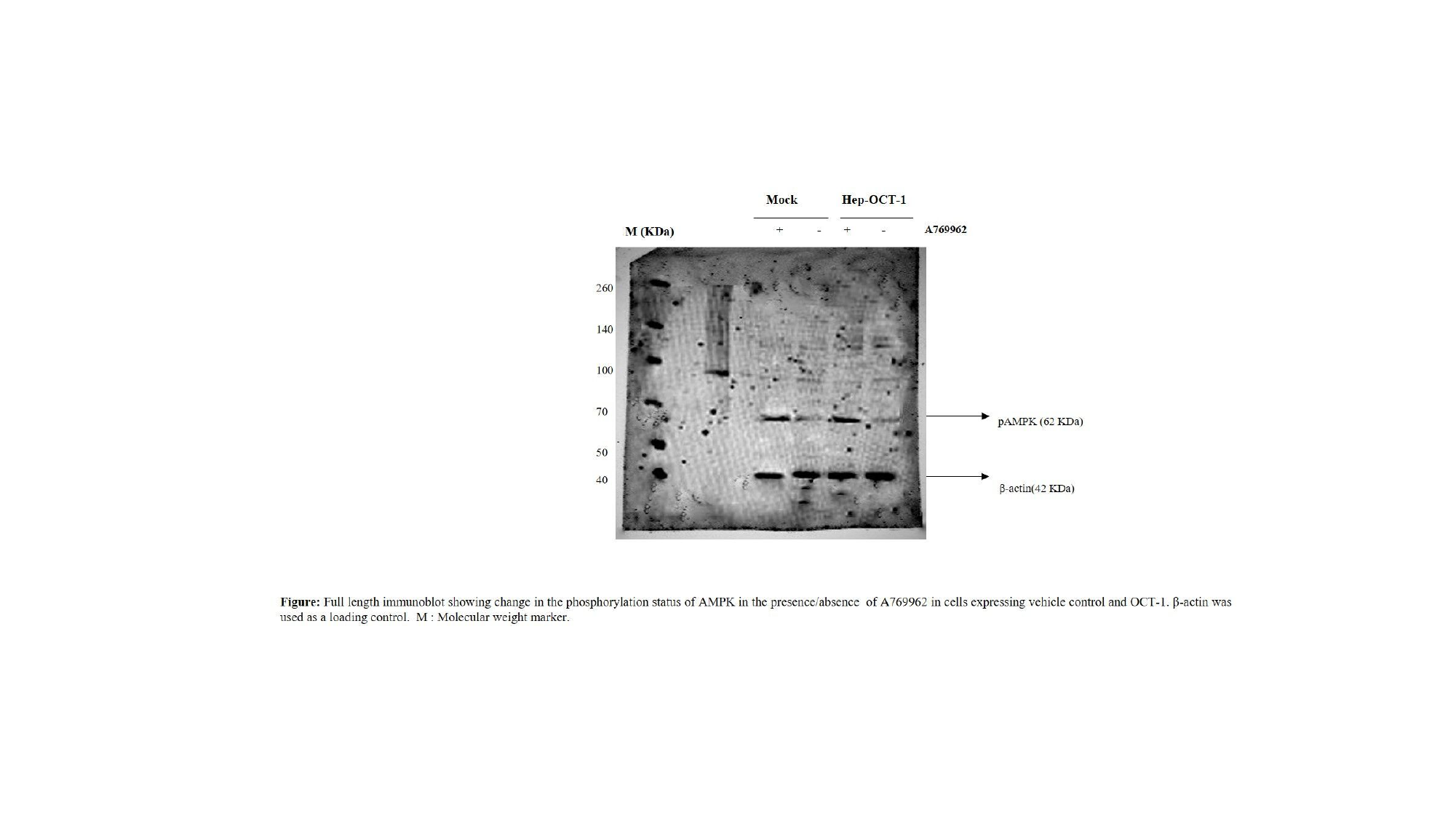

## Slide 2
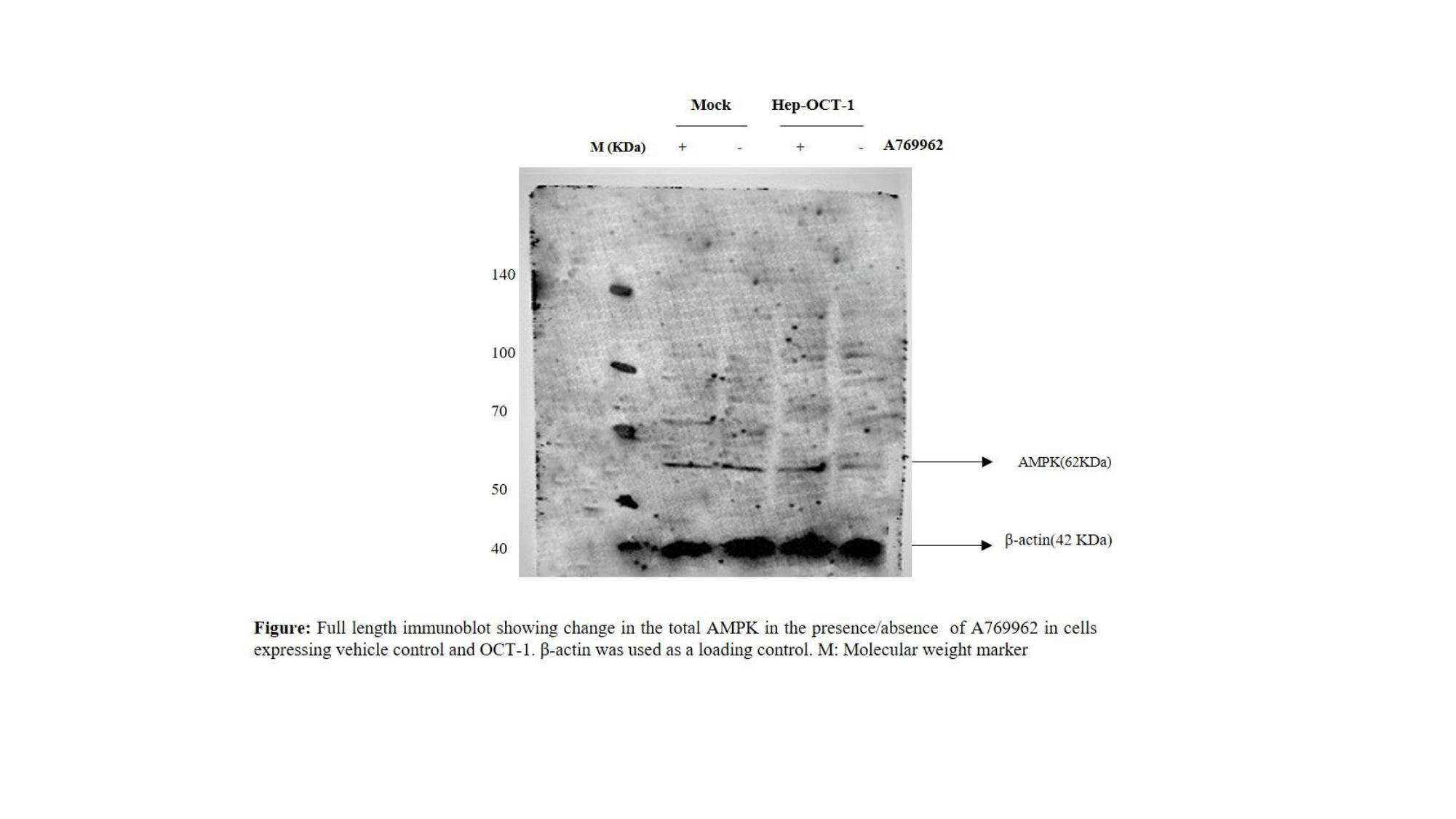

## Slide 3
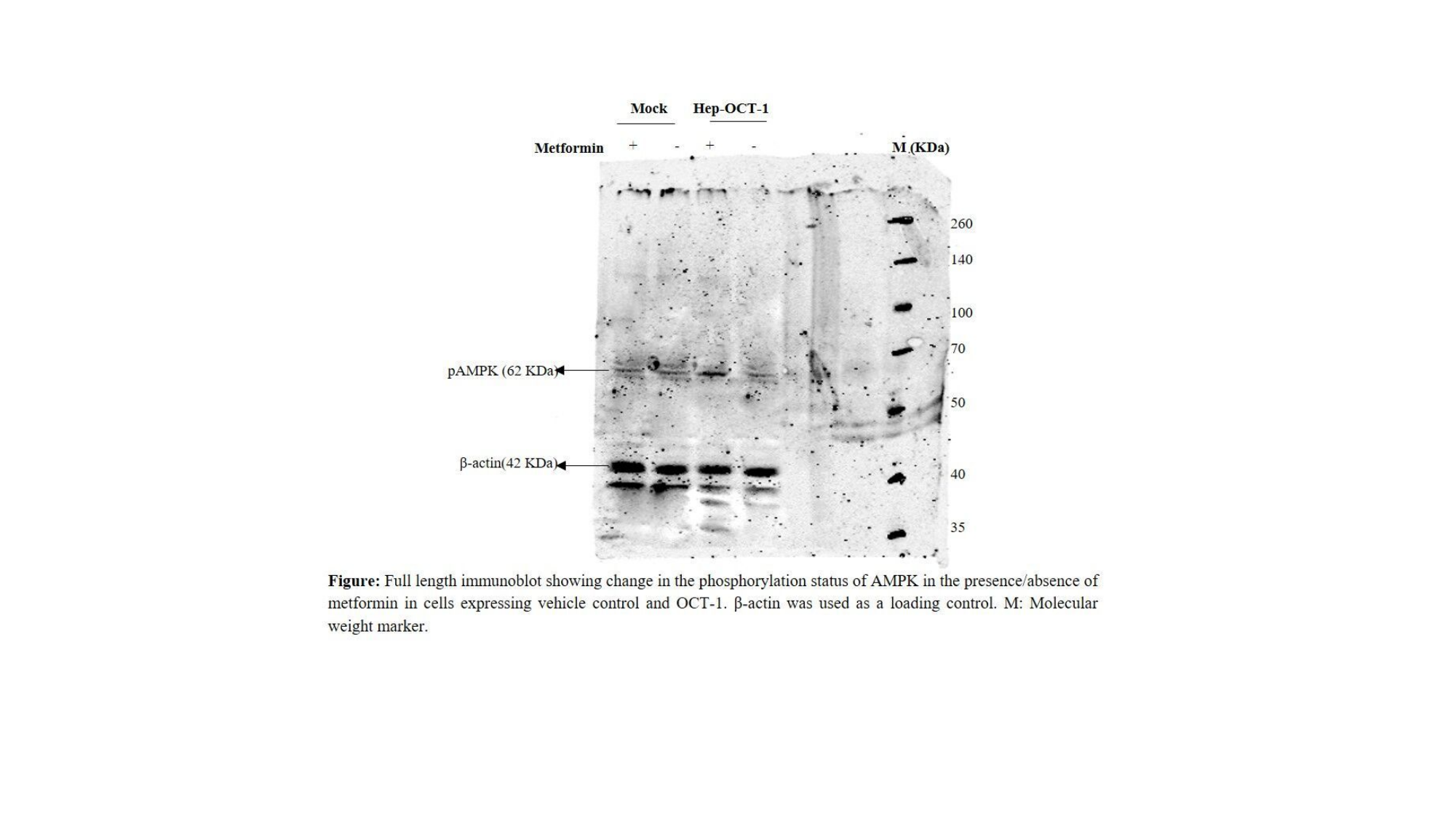

## Slide 4
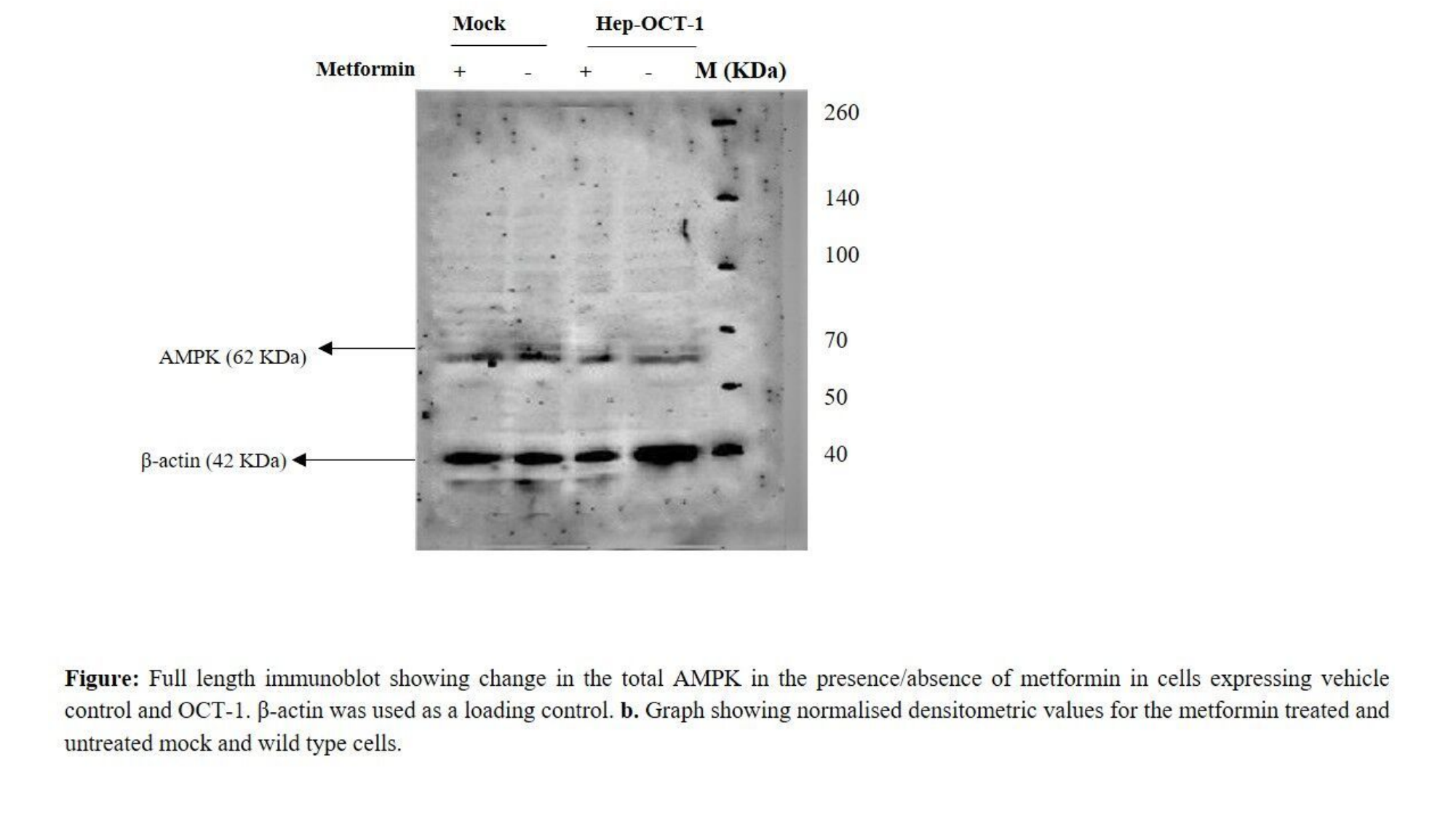

## Slide 5
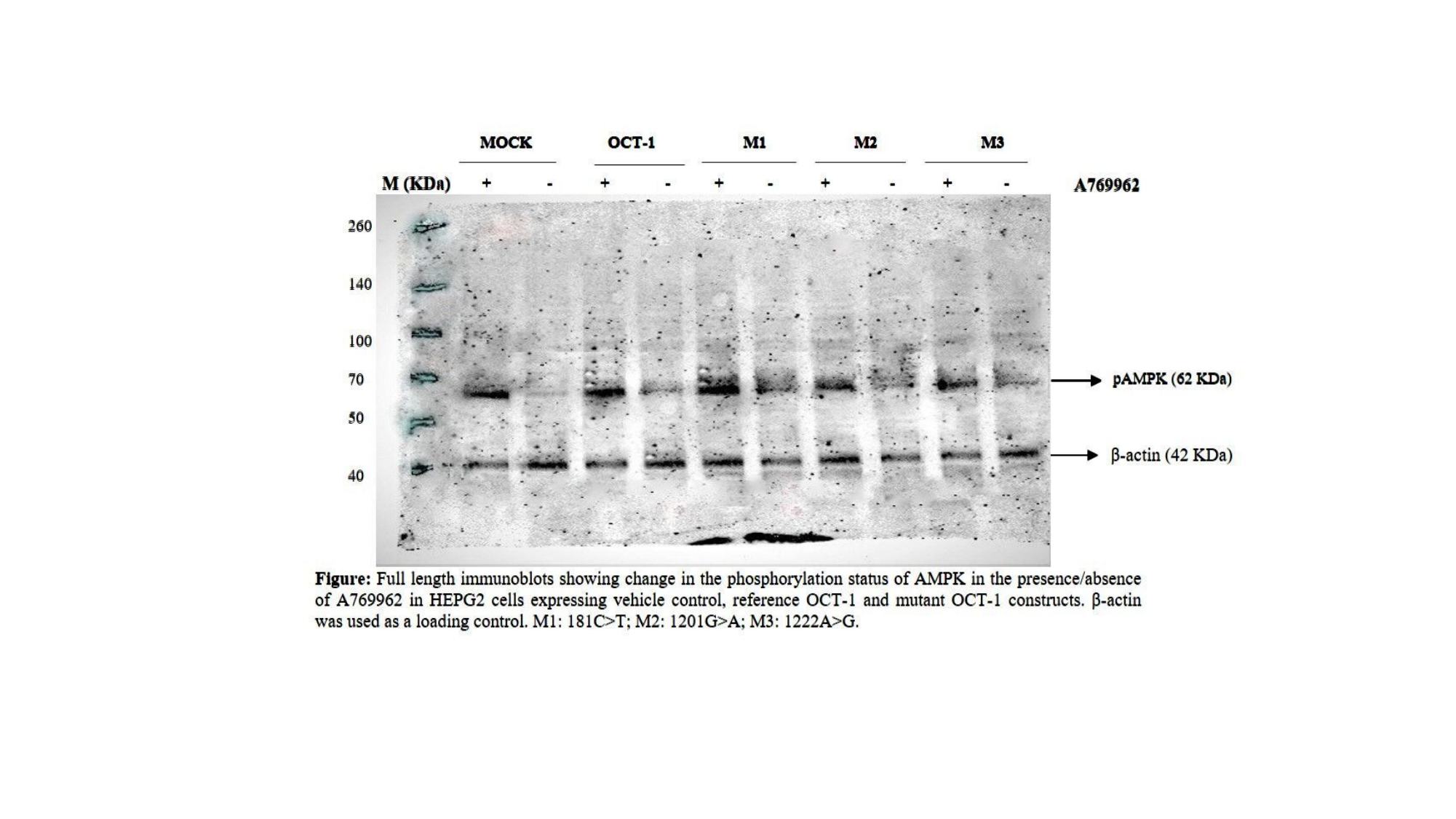

## Slide 6
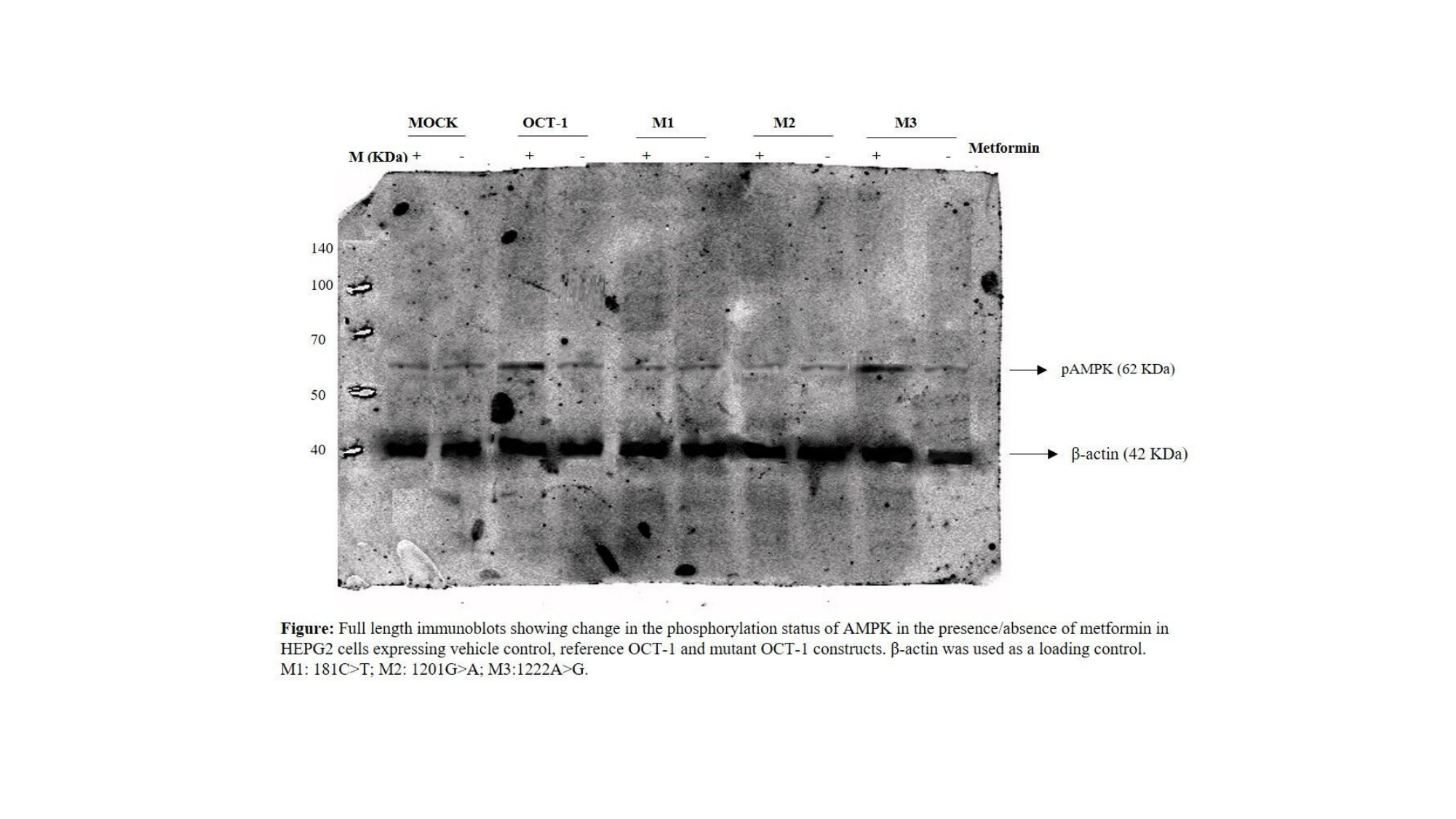

## Slide 7
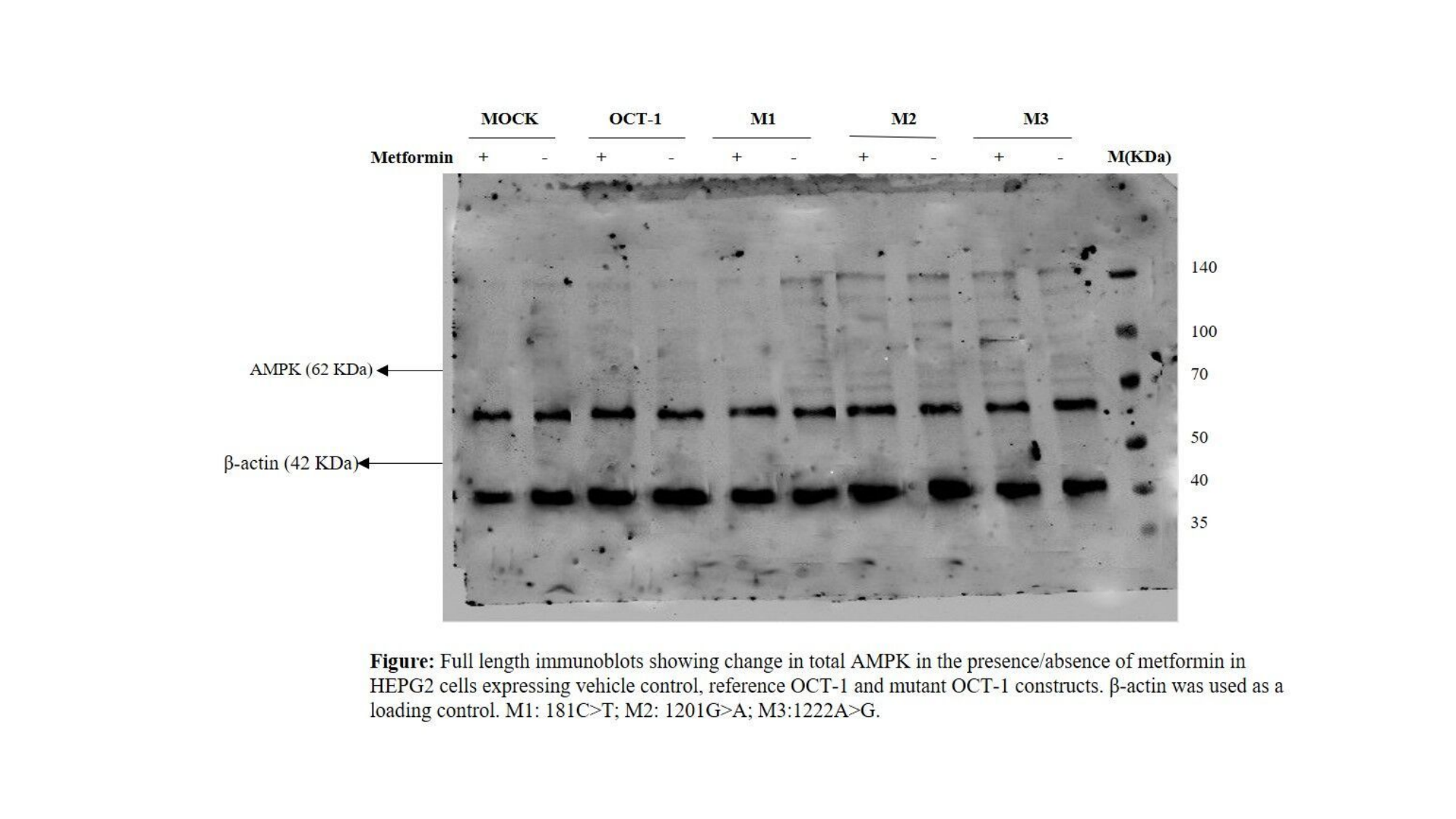

Supplement: Supplementary file 2 — Additional file 2. [file 12902_2022_1033_MOESM2_ESM.pptx]
